# Supplementary material for: Policy stringency during the COVID-19 pandemic and healthcare services utilization in China: An interrupted time-series analysis
Source: PLoS Med. 2026 Mar 26;23(3):e1004672. doi: 10.1371/journal.pmed.1004672 (PMC13043060; doi:10.1371/journal.pmed.1004672)
Supplement: S1 Table — (DOCX) [file pmed.1004672.s001.docx]

**S1 Table** Population and Hospital-based healthcare utilization by regions

| Region | Population | Outpatient Visits | | | | | | | | | |  | Inpatients Discharged | | | | | | | | | |
| --- | --- | --- | --- | --- | --- | --- | --- | --- | --- | --- | --- | --- | --- | --- | --- | --- | --- | --- | --- | --- | --- | --- |
|  | (million) | 2015 | 2016 | 2017 | 2018 | 2019 | 2020 | 2021 | 2022 | 2023 | 2024  (January- April) |  | 2015 | 2016 | 2017 | 2018 | 2019 | 2020 | 2021 | 2022 | 2023 | 2024  (January- April) |
| Anhui | 61.2 | 8952 | 9048 | 9913 | 10342 | 11253 | 12034 | 15027 | 15795 | 14953 | 5319 |  | 624 | 638 | 735 | 747 | 759 | 794 | 835 | 855 | 999 | 359 |
| Beijing | 21.9 | 14010 | 15121 | 14362 | 14722 | 15061 | 10500 | 15220 | 13547 | 16307 | 5440 |  | 261 | 291 | 312 | 338 | 362 | 238 | 335 | 332 | 431 | 151 |
| Chongqing | 31.9 | 5732 | 6283 | 6737 | 7114 | 7795 | 7293 | 9096 | 8245 | 8672 | 3153 |  | 375 | 419 | 456 | 481 | 507 | 453 | 499 | 511 | 587 | 214 |
| Fujian | 41.8 | 8700 | 9092 | 9478 | 9841 | 10004 | 9355 | 11251 | 10730 | 11362 | 4071 |  | 386 | 400 | 426 | 455 | 455 | 453 | 479 | 499 | 578 | 205 |
| Gansu | 24.7 | 3852 | 4401 | 4496 | 4776 | 5016 | 4679 | 6003 | 5401 | 6130 | 2141 |  | 269 | 303 | 308 | 358 | 377 | 342 | 338 | 343 | 467 | 175 |
| Guangdong | 127.1 | 35211 | 36447 | 37148 | 37589 | 39597 | 33475 | 42822 | 37052 | 40762 | 14414 |  | 1105 | 1196 | 1295 | 1371 | 1450 | 1255 | 1409 | 1436 | 1668 | 578 |
| Guangxi | 50.3 | 8577 | 9224 | 9854 | 10304 | 10942 | 9939 | 12278 | 11703 | 11999 | 4222 |  | 490 | 524 | 566 | 601 | 658 | 628 | 686 | 725 | 836 | 295 |
| Guizhou | 38.7 | 4957 | 5664 | 6428 | 7153 | 7558 | 7229 | 8770 | 8421 | 9222 | 3413 |  | 451 | 499 | 569 | 632 | 645 | 618 | 668 | 689 | 781 | 286 |
| Hainan | 10.4 | 1546 | 1612 | 1709 | 1962 | 2094 | 1929 | 2441 | 2154 | 2525 | 949 |  | 79 | 84 | 87 | 101 | 105 | 100 | 111 | 106 | 129 | 46 |
| Hebei | 73.9 | 10930 | 11942 | 12933 | 14320 | 15116 | 15002 | 19280 | 18122 | 19650 | 6716 |  | 708 | 807 | 849 | 966 | 916 | 862 | 880 | 912 | 1111 | 393 |
| Heilongjiang | 30.6 | 5987 | 6274 | 6457 | 6487 | 6703 | 4986 | 6814 | 6339 | 7679 | 2676 |  | 430 | 468 | 502 | 512 | 532 | 316 | 407 | 448 | 618 | 207 |
| Henan | 98.2 | 16775 | 17843 | 18950 | 20494 | 22203 | 20088 | 24368 | 23645 | 25500 | 8875 |  | 1068 | 1158 | 1265 | 1426 | 1524 | 1406 | 1488 | 1486 | 1846 | 662 |
| Hubei | 58.4 | 10920 | 10743 | 12477 | 13925 | 14018 | 11538 | 15527 | 14586 | 15834 | 5468 |  | 723 | 737 | 836 | 915 | 911 | 691 | 885 | 927 | 1090 | 386 |
| Hunan | 65.7 | 8801 | 9279 | 10103 | 10566 | 11387 | 10154 | 12726 | 12734 | 13057 | 4468 |  | 806 | 883 | 957 | 1007 | 1072 | 997 | 1046 | 1031 | 1127 | 399 |
| Jiangsu | 85.3 | 23759 | 24056 | 25939 | 26431 | 26411 | 23886 | 28421 | 25648 | 30179 | 10454 |  | 984 | 1053 | 1115 | 1152 | 1154 | 1087 | 1176 | 1194 | 1407 | 491 |
| Jiangxi | 45.2 | 5743 | 6136 | 6660 | 7233 | 8035 | 7416 | 9545 | 9273 | 9569 | 3539 |  | 436 | 473 | 515 | 565 | 604 | 575 | 626 | 627 | 724 | 265 |
| Jilin | 23.4 | 4920 | 5009 | 5181 | 5325 | 5682 | 4640 | 6508 | 5539 | 6776 | 2253 |  | 311 | 324 | 347 | 355 | 368 | 276 | 323 | 294 | 405 | 139 |
| Liaoning | 41.8 | 8923 | 9443 | 9919 | 10186 | 10633 | 9110 | 11820 | 10423 | 12371 | 4294 |  | 558 | 603 | 641 | 666 | 636 | 521 | 565 | 577 | 733 | 250 |
| Inner Mongolia | 24.0 | 4208 | 4591 | 4849 | 5183 | 5501 | 4951 | 6522 | 6025 | 6541 | 2261 |  | 239 | 268 | 297 | 334 | 320 | 257 | 277 | 277 | 359 | 133 |
| Ningxia | 7.3 | 1715 | 1847 | 1963 | 2073 | 2153 | 1967 | 2427 | 2157 | 2557 | 872 |  | 88 | 97 | 100 | 106 | 108 | 96 | 100 | 101 | 128 | 46 |
| Qinghai | 5.9 | 1067 | 1081 | 1131 | 1312 | 1354 | 1385 | 1818 | 1405 | 1507 | 515 |  | 69 | 74 | 79 | 88 | 96 | 93 | 91 | 87 | 106 | 40 |
| Shaanxi | 39.5 | 7077 | 7717 | 8419 | 8981 | 9838 | 8762 | 11279 | 10228 | 11966 | 4006 |  | 503 | 556 | 614 | 673 | 689 | 589 | 640 | 658 | 826 | 296 |
| Shandong | 101.2 | 18213 | 19807 | 21755 | 23557 | 24385 | 22034 | 27450 | 25111 | 27666 | 9806 |  | 1130 | 1268 | 1380 | 1452 | 1464 | 1330 | 1484 | 1505 | 1862 | 671 |
| Shanghai | 24.9 | 14349 | 14934 | 15330 | 16001 | 16758 | 13288 | 17059 | 14509 | 16670 | 5810 |  | 310 | 339 | 368 | 399 | 447 | 362 | 430 | 343 | 511 | 180 |
| Shanxi | 34.7 | 4411 | 5181 | 5747 | 6216 | 6342 | 6215 | 7974 | 7311 | 8361 | 2817 |  | 288 | 356 | 384 | 421 | 422 | 368 | 395 | 393 | 477 | 171 |
| Sichuan | 83.7 | 15558 | 16699 | 18403 | 19711 | 21774 | 19868 | 24918 | 23796 | 25997 | 9243 |  | 1015 | 1103 | 1217 | 1272 | 1369 | 1235 | 1343 | 1383 | 1638 | 604 |
| Tianjin | 13.6 | 7006 | 7284 | 7049 | 6906 | 6979 | 5397 | 6758 | 5864 | 6991 | 2324 |  | 137 | 150 | 148 | 155 | 161 | 127 | 158 | 162 | 214 | 77 |
| Tibet | 3.7 | 420 | 426 | 445 | 516 | 533 | 545 | 678 | 563 | 628 | 219 |  | 17 | 18 | 19 | 21 | 23 | 23 | 25 | 21 | 26 | 10 |
| Xinjiang | 26.0 | 4414 | 4805 | 4867 | 4823 | 5216 | 5168 | 6716 | 5611 | 7717 | 2740 |  | 362 | 385 | 392 | 385 | 418 | 343 | 398 | 403 | 600 | 229 |
| Yunnan | 46.7 | 8724 | 9426 | 10256 | 11079 | 11737 | 11076 | 13581 | 12702 | 13245 | 4678 |  | 565 | 624 | 690 | 768 | 799 | 768 | 796 | 811 | 915 | 321 |
| Zhejiang | 66.3 | 24028 | 24774 | 27230 | 28989 | 29259 | 26105 | 31539 | 30559 | 32663 | 11201 |  | 706 | 769 | 838 | 916 | 936 | 865 | 1013 | 1042 | 1203 | 421 |
| Total | 1407.7 | 299484 | 316188 | 336188 | 354117 | 371337 | 330012 | 416636 | 385193 | 425057 | 148357 |  | 15492 | 16867 | 18307 | 19639 | 20285 | 18065 | 19904 | 20176 | 24401 | 8696 |
