# Supplementary material for: Policy stringency during the COVID-19 pandemic and healthcare services utilization in China: An interrupted time-series analysis
Source: PLoS Med. 2026 Mar 26;23(3):e1004672. doi: 10.1371/journal.pmed.1004672 (PMC13043060; doi:10.1371/journal.pmed.1004672)
Supplement: S2 Table — (DOCX) [file pmed.1004672.s002.docx]

**S2 Table** Region-specific Policy Stringency Index by different pandemic periods

| Region | The peak of the first wave  (February 2020-March 2020) | The recovery period (April 2020-July 2020) | The period with low COVID transmission in China (August 2020-March 2022) | The Shanghai Outbreak (April 2020-May 2022) | The Omicron wave (June 2022-November 2022 | The lifting of Zero-COVID policy (December 2022- January 2023) | Post Zero-COVID period (February 2023- April 2024) |
| --- | --- | --- | --- | --- | --- | --- | --- |
| Anhui | 70.2 | 49.8 | 50.9 | 71.2 | 70.6 | 24.3 | 1.7 |
| Beijing | 76.7 | 70.0 | 53.5 | 75.7 | 80.4 | 40.2 | 2.2 |
| Chongqing | 67.1 | 58.6 | 49.4 | 52.6 | 63.7 | 28.7 | 3.4 |
| Fujian | 71.8 | 59.8 | 50.9 | 58.0 | 54.6 | 43.4 | 7.9 |
| Gansu | 65.4 | 48.5 | 47.3 | 66.7 | 70.7 | 33.1 | 3.6 |
| Guangdong | 73.4 | 64.5 | 50.6 | 63.4 | 68.5 | 48.1 | 7.9 |
| Guangxi | 73.5 | 48.5 | 45.1 | 55.6 | 59.9 | 38.1 | 2.9 |
| Guizhou | 75.2 | 51.2 | 47.6 | 51.9 | 54.1 | 30.3 | 4.5 |
| Hainan | 59.6 | 43.8 | 38.2 | 57.3 | 52.0 | 20.9 | 2.2 |
| Hebei | 77.3 | 59.9 | 46.1 | 81.0 | 50.2 | 38.2 | 4.0 |
| Heilongjiang | 84.8 | 74.7 | 61.4 | 56.9 | 48.0 | 33.6 | 2.2 |
| Henan | 77.7 | 63.3 | 51.6 | 62.5 | 68.8 | 52.7 | 3.0 |
| Hubei | 86.8 | 69.8 | 53.2 | 57.0 | 59.7 | 36.5 | 5.3 |
| Hunan | 68.1 | 47.5 | 42.3 | 60.3 | 54.5 | 36.0 | 4.3 |
| Jiangsu | 66.2 | 48.1 | 49.9 | 53.2 | 54.5 | 44.2 | 6.9 |
| Jiangxi | 77.1 | 57.2 | 50.8 | 60.5 | 57.1 | 34.2 | 3.0 |
| Jilin | 81.2 | 54.7 | 50.5 | 56.6 | 61.8 | 44.3 | 4.0 |
| Liaoning | 71.8 | 71.0 | 54.8 | 76.2 | 60.4 | 21.7 | 1.7 |
| Inner Mongolia | 69.9 | 47.6 | 45.8 | 81.3 | 65.8 | 34.8 | 4.2 |
| Ningxia | 73.2 | 61.4 | 52.5 | 56.9 | 63.9 | 37.1 | 6.2 |
| Qinghai | 74.4 | 55.4 | 52.7 | 61.1 | 61.9 | 29.8 | 2.3 |
| Shaanxi | 72.3 | 59.5 | 54.2 | 64.2 | 65.5 | 35.7 | 3.6 |
| Shandong | 78.4 | 55.0 | 45.9 | 71.9 | 75.2 | 56.6 | 8.5 |
| Shanghai | 70.5 | 63.3 | 50.7 | 92.7 | 54.5 | 27.3 | 2.9 |
| Shanxi | 73.0 | 58.0 | 51.4 | 67.1 | 69.1 | 35.2 | 2.9 |
| Sichuan | 71.0 | 45.9 | 50.7 | 64.9 | 58.1 | 39.9 | 5.8 |
| Tianjin | 80.2 | 67.0 | 48.6 | 70.9 | 69.7 | 53.1 | 2.9 |
| Tibet | 72.8 | 60.8 | 47.4 | 48.3 | 63.9 | 35.6 | 5.3 |
| Xinjiang | 77.0 | 53.8 | 50.5 | 55.6 | 68.6 | 36.0 | 2.9 |
| Yunnan | 67.4 | 60.9 | 61.5 | 60.3 | 58.5 | 50.5 | 11.2 |
| Zhejiang | 69.6 | 51.7 | 53.9 | 68.6 | 58.0 | 27.7 | 2.8 |
| National | 78.3 | 73.0 | 73.8 | 79.0 | 79.2 | 53.5 | 4.3 |
