# Supplementary material for: Policy stringency during the COVID-19 pandemic and healthcare services utilization in China: An interrupted time-series analysis
Source: PLoS Med. 2026 Mar 26;23(3):e1004672. doi: 10.1371/journal.pmed.1004672 (PMC13043060; doi:10.1371/journal.pmed.1004672)
Supplement: S3 Table — (DOCX) [file pmed.1004672.s003.docx]

**S3 Table** The recovery of healthcare utilizations as of April 2024

|  | **Blow the expected level as of April 2024** | |
| --- | --- | --- |
|  | **Outpatient visits** | **Hospitalizations** |
| Anhui | No | No |
| Beijing | No | Yes |
| Chongqing | Yes | Yes |
| Fujian | No | No |
| Gansu | Yes | Yes |
| Guangdong | No | Yes |
| Guangxi | Yes | No |
| Guizhou | Yes | Yes |
| Hainan | Yes | Yes |
| Hebei | Yes | Yes |
| Heilongjiang | No | Yes |
| Henan | Yes | Yes |
| Hubei | Yes | Yes |
| Hunan | Yes | Yes |
| Inner Mongolia | Yes | Yes |
| Jiangsu | No | No |
| Jiangxi | Yes | Yes |
| Jilin | No | Yes |
| Liaoning | No | Yes |
| Ningxia | Yes | Yes |
| Qinghai | Yes | Yes |
| Shaanxi | Yes | Yes |
| Shandong | Yes | No |
| Shanghai | Yes | Yes |
| Shanxi | Yes | Yes |
| Sichuan | Yes | Yes |
| Tianjin | No | No |
| Tibet | No | Yes |
| Xinjiang | No | No |
| Yunnan | Yes | Yes |
| Zhejiang | Yes | No |
| Total | Yes | Yes |
