# Supplementary material for: Policy stringency during the COVID-19 pandemic and healthcare services utilization in China: An interrupted time-series analysis
Source: PLoS Med. 2026 Mar 26;23(3):e1004672. doi: 10.1371/journal.pmed.1004672 (PMC13043060; doi:10.1371/journal.pmed.1004672)
Supplement: S4 Table — (DOCX) [file pmed.1004672.s004.docx]

**S4 Table** Relative and absolute change in outpatient visits by regions and periods

| Region | The peak of the first wave  (February 2020-March 2020) | | | The recovery period (April 2020-July 2020) | | | The period with low COVID transmission in China (August 2020-March 2022） | | | The Shanghai Outbreak (April 2020-May 2022) | | | The Omicron wave (June 2022-November 2022 | | | The lifting of Zero-COVID policy (December 2022- January 2023) | | | | Post Zero-COVID period (February 2023- April 2024) | | |
| --- | --- | --- | --- | --- | --- | --- | --- | --- | --- | --- | --- | --- | --- | --- | --- | --- | --- | --- | --- | --- | --- | --- |
|  | IRR (96% CI) | Difference (95% CI) * | P-value | IRR (96% CI) | Difference (95% CI) * | P-value | IRR (96% CI) | Difference (95% CI) * | P-value | IRR (96% CI) | Difference (95% CI) * | P-value | IRR (96% CI) | Difference (95% CI) * | P-value | IRR (96% CI) | Difference (95% CI) * | P-value | IRR (96% CI) | | Difference (95% CI) * | P-value |
| Anhui | 0.66 (0.59, 0.74) | -6.93 (-9.51, -4.64) | <.0001 | 1.00 (0.96, 1.05) | -0.02 (-1.89, 1.80) | 0.98 | 1.17 (1.11, 1.24) | 35.38 (23.54, 46.93) | <.0001 | 1.08 (1.01, 1.17) | 1.88 (0.17, 3.55) | 0.03 | 1.19 (1.11, 1.27) | 12.88 (8.33, 17.21) | <.0001 | 1.01 (0.81, 1.20) | -0.08 (-5.06, 3.74) | 0.97 | 1.04 (0.95, 1.14) | | 7.51 (-9.61, 24.09) | 0.370 |
| Beijing | 0.32 (0.30, 0.34) | -17.12 (-18.69, -15.58) | <.0001 | 0.63 (0.61, 0.65) | -19.04 (-20.63, -17.52) | <.0001 | 0.96 (0.93, 0.99) | -10.10 (-18.29, -2.50) | 0.01 | 0.81 (0.78, 0.85) | -4.91 (-6.09, -3.77) | <.0001 | 0.90 (0.86, 0.94) | -7.69 (-11.09, -4.60) | <.0001 | 0.81 (0.75, 0.87) | -4.39 (-6.00, -2.80) | <.0001 | 1.07 (1.02, 1.13) | | 14.28 (4.62, 23.36) | 0.004 |
| Chongqing | 0.57 (0.55, 0.60) | -5.85 (-6.48, -5.27) | <.0001 | 0.88 (0.86, 0.90) | -3.38 (-4.05, -2.75) | <.0001 | 1.00 (0.97, 1.02) | -0.28 (-4.36, 3.50) | 0.93 | 0.92 (0.88, 0.95) | -1.34 (-1.96, -0.76) | <.0001 | 0.83 (0.80, 0.86) | -8.25 (-9.79, -6.76) | <.0001 | 0.76 (0.72, 0.81) | -3.66 (-4.57, -2.74) | <.0001 | 0.88 (0.84, 0.92) | | -15.56 (-21.26, -10.19) | <.0001 |
| Fujian | 0.63 (0.59, 0.66) | -6.17 (-7.14, -5.27) | <.0001 | 0.87 (0.85, 0.90) | -4.79 (-5.90, -3.69) | <.0001 | 1.01 (0.98, 1.05) | 2.28 (-3.65, 8.11) | 0.47 | 0.87 (0.83, 0.92) | -2.53 (-3.46, -1.61) | <.0001 | 0.99 (0.94, 1.03) | -0.90 (-3.46, 1.78) | 0.50 | 0.82 (0.77, 0.87) | -3.10 (-4.18, -2.08) | <.0001 | 1.02 (0.97, 1.07) | | 2.56 (-5.12, 10.15) | 0.528 |
| Gansu | 0.67 (0.64, 0.71) | -2.97 (-3.44, -2.50) | <.0001 | 0.91 (0.89, 0.94) | -1.56 (-2.11, -0.97) | <.0001 | 1.02 (0.99, 1.05) | 1.61 (-1.16, 4.29) | 0.27 | 0.98 (0.94, 1.03) | -0.17 (-0.61, 0.27) | 0.47 | 0.82 (0.78, 0.85) | -5.58 (-7.11, -4.17) | <.0001 | 0.82 (0.78, 0.88) | -1.82 (-2.46, -1.21) | <.0001 | 0.97 (0.93, 1.02) | | -2.30 (-6.28, 1.64) | 0.292 |
| Guangdong | 0.51 (0.49, 0.53) | -30.91 (-33.28, -28.57) | <.0001 | 0.78 (0.76, 0.81) | -30.55 (-34.10, -26.67) | <.0001 | 1.01 (0.99, 1.04) | 9.39 (-9.73, 27.82) | 0.30 | 0.88 (0.84, 0.91) | -9.06 (-12.10, -6.08) | <.0001 | 0.93 (0.89, 0.96) | -15.75 (-23.21, -8.67) | <.0001 | 0.76 (0.72, 0.80) | -14.69 (-17.83, -11.74) | <.0001 | 1.02 (0.97, 1.06) | | 7.77 (-15.31, 29.87) | 0.482 |
| Guangxi | 0.59 (0.57, 0.61) | -7.61 (-8.33, -6.97) | <.0001 | 0.82 (0.80, 0.84) | -7.18 (-8.18, -6.20) | <.0001 | 0.98 (0.95, 1.00) | -5.07 (-10.29, 0.20) | 0.06 | 0.89 (0.86, 0.92) | -2.50 (-3.31, -1.71) | <.0001 | 0.91 (0.88, 0.94) | -5.84 (-7.99, -3.85) | <.0001 | 0.79 (0.75, 0.83) | -4.23 (-5.19, -3.19) | <.0001 | 0.90 (0.86, 0.93) | | -18.17 (-24.96, -11.36) | <.0001 |
| Guizhou | 0.62 (0.59, 0.64) | -5.39 (-5.96, -4.84) | <.0001 | 0.82 (0.79, 0.85) | -5.35 (-6.50, -4.24) | <.0001 | 0.92 (0.88, 0.96) | -13.19 (-19.96, -6.40) | <.0001 | 0.81 (0.77, 0.85) | -3.43 (-4.36, -2.53) | <.0001 | 0.78 (0.73, 0.82) | -12.05 (-15.10, -9.01) | <.0001 | 0.75 (0.70, 0.80) | -4.28 (-5.37, -3.22) | <.0001 | 0.81 (0.76, 0.86) | | -28.43 (-37.68, -18.87) | <.0001 |
| Hainan | 0.52 (0.49, 0.56) | -1.82 (-2.08, -1.59) | <.0001 | 0.83 (0.81, 0.86) | -1.32 (-1.57, -1.09) | <.0001 | 0.98 (0.93, 1.02) | -0.91 (-2.97, 0.95) | 0.36 | 0.82 (0.77, 0.87) | -0.84 (-1.14, -0.56) | <.0001 | 0.77 (0.72, 0.81) | -3.11 (-3.90, -2.39) | <.0001 | 0.75 (0.69, 0.81) | -1.06 (-1.40, -0.73) | <.0001 | 0.89 (0.83, 0.96) | | -3.96 (-6.84, -1.40) | 0.006 |
| Hebei | 0.59 (0.56, 0.63) | -10.85 (-12.58, -9.14) | <.0001 | 0.89 (0.86, 0.92) | -6.36 (-8.13, -4.56) | <.0001 | 1.05 (1.01, 1.09) | 14.71 (4.35, 25.82) | 0.01 | 0.88 (0.84, 0.93) | -3.96 (-5.50, -2.30) | <.0001 | 0.93 (0.89, 0.97) | -6.75 (-11.15, -2.54) | <.0001 | 0.84 (0.80, 0.89) | -5.02 (-6.62, -3.38) | <.0001 | 0.94 (0.89, 1.00) | | -16.19 (-31.81, -0.14) | 0.046 |
| Heilongjiang | 0.37 (0.35, 0.39) | -7.57 (-8.22, -6.99) | <.0001 | 0.64 (0.63, 0.65) | -8.41 (-8.83, -8.03) | <.0001 | 0.97 (0.95, 0.99) | -3.63 (-6.10, -0.94) | 0.01 | 0.76 (0.73, 0.78) | -2.99 (-3.36, -2.63) | <.0001 | 0.90 (0.88, 0.93) | -3.45 (-4.34, -2.52) | <.0001 | 1.02 (0.95, 1.09) | 0.15 (-0.62, 0.95) | 0.70 | 1.04 (1.00, 1.07) | | 3.43 (0.31, 6.82) | 0.032 |
| Henan | 0.56 (0.54, 0.58) | -17.24 (-18.43, -16.11) | <.0001 | 0.86 (0.85, 0.88) | -11.14 (-12.43, -9.82) | <.0001 | 0.97 (0.95, 0.99) | -12.11 (-19.89, -4.45) | <.0001 | 0.88 (0.85, 0.90) | -5.66 (-6.95, -4.35) | <.0001 | 0.85 (0.83, 0.87) | -20.19 (-23.53, -16.83) | <.0001 | 0.86 (0.83, 0.89) | -5.83 (-7.36, -4.30) | <.0001 | 0.91 (0.88, 0.93) | | -34.11 (-45.35, -23.14) | <.0001 |
| Hubei | 0.31 (0.29, 0.34) | -16.84 (-18.83, -14.89) | <.0001 | 0.73 (0.69, 0.76) | -14.46 (-17.07, -12.00) | <.0001 | 0.94 (0.89, 0.99) | -15.89 (-30.93, -1.77) | 0.03 | 0.86 (0.80, 0.93) | -4.17 (-6.55, -1.82) | <.0001 | 0.81 (0.76, 0.86) | -17.55 (-23.25, -11.97) | <.0001 | 0.76 (0.71, 0.82) | -6.50 (-8.58, -4.53) | <.0001 | 0.85 (0.78, 0.93) | | -35.23 (-57.53, -14.17) | <.0001 |
| Hunan | 0.53 (0.51, 0.56) | -9.37 (-10.28, -8.49) | <.0001 | 0.85 (0.83, 0.87) | -6.46 (-7.54, -5.42) | <.0001 | 0.98 (0.95, 1.01) | -3.74 (-10.77, 2.81) | 0.26 | 0.89 (0.86, 0.93) | -2.55 (-3.54, -1.59) | <.0001 | 0.95 (0.92, 0.99) | -3.46 (-6.03, -0.98) | <.0001 | 0.85 (0.78, 0.92) | -3.21 (-5.07, -1.60) | <.0001 | 0.91 (0.87, 0.95) | | -17.00 (-25.73, -8.57) | <.0001 |
| Jiangsu | 0.51 (0.49, 0.54) | -4.97 (-5.49, -4.47) | <.0001 | 0.86 (0.84, 0.88) | -2.83 (-3.31, -2.31) | <.0001 | 1.01 (0.98, 1.05) | 1.18 (-2.34, 4.88) | 0.50 | 0.98 (0.94, 1.03) | -0.24 (-0.71, 0.28) | 0.35 | 0.89 (0.86, 0.93) | -3.72 (-4.92, -2.38) | <.0001 | 0.83 (0.75, 0.91) | -1.84 (-2.80, -0.81) | <.0001 | 0.92 (0.87, 0.96) | | -7.79 (-12.15, -3.21) | <.0001 |
| Jiangxi | 0.57 (0.54, 0.61) | -18.27 (-20.76, -15.98) | <.0001 | 0.84 (0.82, 0.87) | -14.83 (-17.81, -11.88) | <.0001 | 0.98 (0.95, 1.01) | -9.61 (-26.69, 6.42) | 0.25 | 0.77 (0.73, 0.80) | -11.82 (-14.36, -9.42) | <.0001 | 0.91 (0.87, 0.94) | -14.21 (-20.05, -8.66) | <.0001 | 0.80 (0.77, 0.83) | -9.69 (-11.49, -8.05) | <.0001 | 1.04 (0.98, 1.10) | | 12.90 (-9.26, 33.99) | 0.272 |
| Jilin | 0.58 (0.55, 0.61) | -6.03 (-6.85, -5.25) | <.0001 | 0.85 (0.82, 0.88) | -4.35 (-5.41, -3.39) | <.0001 | 1.00 (0.96, 1.04) | 0.22 (-6.25, 6.14) | 0.92 | 0.86 (0.81, 0.91) | -2.47 (-3.48, -1.52) | <.0001 | 0.91 (0.87, 0.96) | -4.43 (-6.87, -2.16) | <.0001 | 0.85 (0.80, 0.90) | -2.40 (-3.34, -1.55) | <.0001 | 0.88 (0.83, 0.94) | | -16.69 (-26.13, -8.16) | <.0001 |
| Liaoning | 0.52 (0.49, 0.54) | -4.83 (-5.33, -4.38) | <.0001 | 0.75 (0.74, 0.77) | -4.85 (-5.32, -4.38) | <.0001 | 1.04 (1.01, 1.08) | 4.25 (0.69, 7.84) | 0.03 | 0.56 (0.54, 0.59) | -4.62 (-5.04, -4.20) | <.0001 | 1.00 (0.95, 1.04) | -0.14 (-1.54, 1.26) | 0.86 | 0.96 (0.90, 1.03) | -0.34 (-0.98, 0.24) | 0.24 | 1.05 (0.99, 1.10) | | 3.66 (-0.98, 8.06) | 0.126 |
| Inner Mongolia | 0.52 (0.50, 0.55) | -8.82 (-9.74, -8.06) | <.0001 | 0.81 (0.80, 0.83) | -7.04 (-7.90, -6.27) | <.0001 | 1.00 (0.97, 1.02) | -0.77 (-5.71, 3.88) | 0.76 | 0.77 (0.74, 0.80) | -4.69 (-5.46, -3.97) | <.0001 | 0.88 (0.86, 0.91) | -7.18 (-9.00, -5.39) | <.0001 | 0.87 (0.80, 0.94) | -2.44 (-3.97, -0.96) | <.0001 | 1.01 (0.97, 1.04) | | 1.27 (-4.78, 6.54) | 0.656 |
| Ningxia | 0.56 (0.53, 0.59) | -1.69 (-1.91, -1.49) | <.0001 | 0.88 (0.85, 0.91) | -0.91 (-1.15, -0.67) | <.0001 | 0.99 (0.95, 1.03) | -0.58 (-2.10, 1.08) | 0.49 | 0.88 (0.84, 0.93) | -0.49 (-0.70, -0.28) | <.0001 | 0.80 (0.77, 0.83) | -2.58 (-3.09, -2.07) | <.0001 | 0.87 (0.81, 0.93) | -0.54 (-0.82, -0.26) | <.0001 | 0.97 (0.91, 1.04) | | -0.95 (-3.05, 1.23) | 0.392 |
| Qinghai | 0.78 (0.70, 0.85) | -0.53 (-0.76, -0.31) | <.0001 | 0.94 (0.89, 0.99) | -0.31 (-0.58, -0.06) | 0.02 | 1.14 (1.07, 1.21) | 3.58 (1.98, 4.98) | <.0001 | 0.86 (0.78, 0.93) | -0.38 (-0.64, -0.17) | <.0001 | 0.76 (0.71, 0.81) | -2.04 (-2.65, -1.45) | <.0001 | 0.79 (0.73, 0.85) | -0.57 (-0.76, -0.38) | <.0001 | 0.87 (0.79, 0.96) | | -2.81 (-5.02, -0.88) | <.0001 |
| Shaanxi | 0.50 (0.48, 0.52) | -8.86 (-9.52, -8.24) | <.0001 | 0.84 (0.83, 0.85) | -5.70 (-6.19, -5.23) | <.0001 | 0.95 (0.93, 0.96) | -9.83 (-12.67, -7.09) | <.0001 | 0.92 (0.90, 0.94) | -1.61 (-2.08, -1.13) | <.0001 | 0.84 (0.82, 0.85) | -10.19 (-11.42, -8.97) | <.0001 | 0.79 (0.74, 0.83) | -4.22 (-5.29, -3.15) | <.0001 | 0.90 (0.88, 0.92) | | -16.51 (-20.54, -12.61) | <.0001 |
| Shandong | 0.54 (0.52, 0.56) | -20.01 (-21.87, -18.39) | <.0001 | 0.82 (0.79, 0.84) | -16.66 (-19.18, -14.17) | <.0001 | 0.94 (0.91, 0.96) | -29.64 (-43.61, -16.73) | <.0001 | 0.80 (0.77, 0.84) | -10.10 (-12.66, -7.61) | <.0001 | 0.81 (0.78, 0.83) | -30.00 (-34.87, -25.34) | <.0001 | 0.74 (0.70, 0.79) | -12.73 (-15.64, -9.92) | <.0001 | 0.86 (0.82, 0.89) | | -60.23 (-78.81, -43.28) | <.0001 |
| Shanghai | 0.41 (0.39, 0.43) | -17.87 (-19.28, -16.44) | <.0001 | 0.78 (0.76, 0.81) | -12.58 (-14.64, -10.65) | <.0001 | 0.95 (0.92, 0.98) | -15.25 (-25.99, -4.85) | <.0001 | 0.36 (0.34, 0.38) | -19.61 (-21.50, -17.80) | <.0001 | 0.80 (0.77, 0.83) | -18.66 (-22.27, -15.03) | <.0001 | 0.68 (0.62, 0.74) | -9.08 (-11.67, -6.60) | <.0001 | 0.88 (0.83, 0.92) | | -30.43 (-43.45, -18.38) | <.0001 |
| Shanxi | 0.56 (0.52, 0.61) | -5.33 (-6.27, -4.38) | <.0001 | 0.89 (0.84, 0.93) | -2.80 (-4.02, -1.55) | <.0001 | 1.00 (0.94, 1.06) | -0.62 (-8.43, 7.16) | 0.88 | 0.86 (0.79, 0.93) | -2.07 (-3.24, -0.91) | 0.002 | 0.80 (0.75, 0.87) | -8.65 (-11.91, -5.44) | <.0001 | 0.80 (0.71, 0.91) | -2.69 (-4.40, -1.05) | <.0001 | 0.88 (0.81, 0.97) | | -14.40 (-25.69, -3.31) | 0.012 |
| Sichuan | 0.59 (0.57, 0.62) | -15.76 (-17.17, -14.38) | <.0001 | 0.84 (0.82, 0.86) | -12.45 (-14.30, -10.79) | <.0001 | 0.97 (0.94, 0.99) | -14.23 (-26.02, -3.08) | 0.01 | 0.89 (0.86, 0.92) | -5.13 (-6.85, -3.48) | <.0001 | 0.84 (0.81, 0.87) | -22.43 (-27.16, -17.71) | <.0001 | 0.80 (0.76, 0.84) | -9.03 (-11.29, -6.94) | <.0001 | 0.88 (0.85, 0.92) | | -43.88 (-60.95, -27.75) | <.0001 |
| Tianjin | 0.43 (0.41, 0.45) | -6.51 (-6.97, -6.05) | <.0001 | 0.77 (0.75, 0.79) | -5.43 (-6.11, -4.73) | <.0001 | 0.95 (0.92, 0.97) | -6.25 (-9.42, -3.12) | <.0001 | 0.86 (0.82, 0.89) | -1.72 (-2.23, -1.23) | <.0001 | 0.89 (0.85, 0.92) | -4.01 (-5.34, -2.72) | <.0001 | 0.83 (0.79, 0.88) | -1.67 (-2.24, -1.14) | <.0001 | 1.04 (0.99, 1.09) | | 3.31 (-0.80, 7.16) | 0.106 |
| Tibet | 0.77 (0.66, 0.89) | -0.23 (-0.38, -0.10) | <.0001 | 1.06 (0.99, 1.13) | 0.11 (-0.01, 0.23) | 0.08 | 1.14 (1.08, 1.23) | 1.38 (0.79, 2.05) | <.0001 | 1.07 (0.96, 1.18) | 0.06 (-0.04, 0.16) | 0.22 | 0.72 (0.66, 0.78) | -0.88 (-1.14, -0.64) | <.0001 | 1.18 (0.99, 1.37) | 0.15 (-0.01, 0.28) | 0.06 | 0.99 (0.89, 1.10) | | -0.10 (-0.94, 0.73) | 0.820 |
| Xinjiang | 0.66 (0.61, 0.70) | -3.09 (-3.76, -2.48) | <.0001 | 1.08 (1.04, 1.12) | 1.34 (0.66, 1.98) | <.0001 | 1.19 (1.14, 1.23) | 16.96 (13.32, 20.38) | <.0001 | 1.24 (1.17, 1.32) | 2.22 (1.67, 2.74) | <.0001 | 0.81 (0.77, 0.85) | -5.22 (-6.54, -3.90) | <.0001 | 1.08 (1.02, 1.15) | 0.74 (0.21, 1.25) | 0.01 | 1.41 (1.33, 1.51) | | 29.17 (24.67, 33.47) | <.0001 |
| Yunnan | 0.67 (0.64, 0.70) | -6.75 (-7.61, -5.94) | <.0001 | 0.85 (0.84, 0.87) | -6.26 (-7.13, -5.47) | <.0001 | 0.97 (0.95, 0.99) | -6.83 (-12.09, -1.84) | 0.01 | 0.85 (0.82, 0.87) | -3.74 (-4.39, -3.10) | <.0001 | 0.86 (0.83, 0.88) | -10.77 (-13.13, -8.73) | <.0001 | 0.76 (0.72, 0.79) | -5.92 (-7.06, -4.80) | <.0001 | 0.85 (0.82, 0.88) | | -29.85 (-37.10, -22.91) | <.0001 |
| Zhejiang | 0.50 (0.47, 0.52) | -25.75 (-28.24, -23.33) | <.0001 | 0.83 (0.81, 0.85) | -18.29 (-20.96, -15.71) | <.0001 | 0.93 (0.89, 0.96) | -41.28 (-63.00, -21.27) | 0.00 | 0.85 (0.82, 0.88) | -9.04 (-11.44, -6.78) | <.0001 | 0.90 (0.87, 0.94) | -17.39 (-25.10, -10.10) | <.0001 | 0.78 (0.72, 0.85) | -11.46 (-16.04, -7.18) | <.0001 | 0.90 (0.85, 0.95) | | -45.71 (-70.96, -22.08) | <.0001 |
| Total | 0.53 (0.51, 0.55) | -302.57 (-322.92, -282.05) | <.0001 | 0.83 (0.82, 0.84) | -224.69 (-240.42, -209.66) | <.0001 | 1.00 (0.98, 1.01) | -32.41 (-148.06, 82.32) | 0.60 | 0.85 (0.83, 0.87) | -108.53 (-123.92, -93.85) | <.0001 | 0.91 (0.89, 0.93) | -201.83 (-241.04, -160.52) | <.0001 | 0.83 (0.79, 0.87) | -111.75 (-143.95, -79.92) | <.0001 | 0.96 (0.93, 0.98) | | -244.91 (-394.05, -96.11) | 0.004 |

*Measured in millions

Note: Blue cells indicate a statistically significant decrease, while light blue cells represent a decrease that is not statistically significant; Orange cells indicate a statistically significant increase, while light orange cells represent an increase that is not statistically significant
