# Supplementary material for: Policy stringency during the COVID-19 pandemic and healthcare services utilization in China: An interrupted time-series analysis
Source: PLoS Med. 2026 Mar 26;23(3):e1004672. doi: 10.1371/journal.pmed.1004672 (PMC13043060; doi:10.1371/journal.pmed.1004672)
Supplement: S5 Table — (DOCX) [file pmed.1004672.s005.docx]

**S5 Table** Relative and absolute change in hospitalizations by regions and periods

| Region | The peak of the first wave  (February 2020-March 2020) | | | The recovery period (April 2020-July 2020) | | | The period with low COVID transmission in China (August 2020-March 2022） | | | | The Shanghai Outbreak (April 2020-May 2022) | | | | The Omicron wave (June 2022-November 2022 | | | | The lifting of Zero-COVID policy (December 2022- January 2023) | | | | Post Zero-COVID period (February 2023- April 2024) | | |
| --- | --- | --- | --- | --- | --- | --- | --- | --- | --- | --- | --- | --- | --- | --- | --- | --- | --- | --- | --- | --- | --- | --- | --- | --- | --- |
|  | IRR (96% CI) | Difference (95% CI) * | P-value | IRR (96% CI) | Difference (95% CI) * | P-value | IRR (96% CI) | Difference (95% CI) * | P-value | IRR (96% CI) | | Difference (95% CI) * | P-value | IRR (96% CI) | | Difference (95% CI) * | P-value | IRR (96% CI) | | Difference (95% CI) * | P-value | IRR (96% CI) | | Difference (95% CI) * | P-value |
| Anhui | 0.62 (0.56, 0.68) | -0.56 (-0.70, -0.42) | <.0001 | 0.97 (0.92, 1.02) | -0.08 (-0.23, 0.06) | 0.27 | 0.97 (0.93, 1.03) | -0.41 (-1.15, 0.38) | 0.33 | 0.76 (0.71, 0.82) | | -0.39 (-0.50, -0.27) | <.0001 | 0.94 (0.88, 1.02) | | -0.25 (-0.58, 0.07) | 0.14 | 1.08 (0.97, 1.20) | | 0.11 (-0.05, 0.26) | 0.19 | 1.01 (0.94, 1.11) | | 0.17 (-0.87, 1.23) | 0.76 |
| Beijing | 0.26 (0.26, 0.27) | -0.46 (-0.47, -0.45) | <.0001 | 0.52 (0.51, 0.53) | -0.64 (-0.66, -0.62) | <.0001 | 0.78 (0.76, 0.79) | -1.56 (-1.70, -1.43) | <.0001 | 0.65 (0.63, 0.67) | | -0.27 (-0.29, -0.25) | <.0001 | 0.75 (0.73, 0.76) | | -0.59 (-0.64, -0.54) | <.0001 | 0.65 (0.62, 0.68) | | -0.26 (-0.29, -0.23) | <.0001 | 0.89 (0.86, 0.91) | | -0.71 (-0.90, -0.55) | <.0001 |
| Chongqing | 0.56 (0.54, 0.58) | -0.40 (-0.43, -0.37) | <.0001 | 0.84 (0.81, 0.86) | -0.31 (-0.36, -0.26) | <.0001 | 0.85 (0.82, 0.88) | -1.50 (-1.86, -1.16) | <.0001 | 0.81 (0.77, 0.84) | | -0.21 (-0.25, -0.16) | <.0001 | 0.81 (0.77, 0.84) | | -0.61 (-0.76, -0.47) | <.0001 | 0.82 (0.77, 0.88) | | -0.19 (-0.26, -0.13) | <.0001 | 0.89 (0.84, 0.94) | | -0.96 (-1.43, -0.52) | <.0001 |
| Fujian | 0.73 (0.69, 0.78) | -0.20 (-0.25, -0.16) | <.0001 | 0.91 (0.88, 0.94) | -0.16 (-0.21, -0.10) | <.0001 | 0.95 (0.91, 0.99) | -0.40 (-0.76, -0.06) | 0.02 | 0.83 (0.80, 0.88) | | -0.16 (-0.20, -0.11) | <.0001 | 0.98 (0.93, 1.03) | | -0.07 (-0.21, 0.07) | 0.33 | 0.82 (0.76, 0.89) | | -0.15 (-0.22, -0.09) | <.0001 | 1.08 (1.02, 1.15) | | 0.57 (0.12, 0.99) | 0.02 |
| Gansu | 0.62 (0.58, 0.67) | -0.26 (-0.32, -0.22) | <.0001 | 0.87 (0.84, 0.90) | -0.18 (-0.24, -0.14) | <.0001 | 0.78 (0.75, 0.82) | -1.59 (-1.94, -1.24) | <.0001 | 0.71 (0.67, 0.75) | | -0.23 (-0.28, -0.19) | <.0001 | 0.66 (0.62, 0.70) | | -0.80 (-0.94, -0.66) | <.0001 | 0.80 (0.76, 0.85) | | -0.18 (-0.23, -0.13) | <.0001 | 0.90 (0.84, 0.97) | | -0.64 (-1.12, -0.18) | 0.00 |
| Guangdong | 0.57 (0.55, 0.58) | -1.02 (-1.09, -0.95) | <.0001 | 0.78 (0.76, 0.79) | -1.20 (-1.30, -1.10) | <.0001 | 0.86 (0.84, 0.87) | -3.94 (-4.51, -3.40) | <.0001 | 0.80 (0.78, 0.83) | | -0.58 (-0.67, -0.50) | <.0001 | 0.85 (0.83, 0.87) | | -1.38 (-1.60, -1.17) | <.0001 | 0.78 (0.76, 0.81) | | -0.59 (-0.69, -0.49) | <.0001 | 0.93 (0.90, 0.96) | | -1.57 (-2.37, -0.81) | <.0001 |
| Guangxi | 0.70 (0.68, 0.72) | -0.34 (-0.37, -0.30) | <.0001 | 0.88 (0.86, 0.90) | -0.30 (-0.36, -0.24) | <.0001 | 0.92 (0.90, 0.94) | -1.02 (-1.31, -0.74) | <.0001 | 0.85 (0.82, 0.89) | | -0.20 (-0.25, -0.15) | <.0001 | 0.92 (0.90, 0.95) | | -0.32 (-0.44, -0.21) | <.0001 | 0.97 (0.94, 1.00) | | -0.04 (-0.08, 0.00) | 0.07 | 0.99 (0.95, 1.03) | | -0.10 (-0.51, 0.27) | 0.61 |
| Guizhou | 0.68 (0.65, 0.71) | -0.38 (-0.44, -0.33) | <.0001 | 0.84 (0.80, 0.88) | -0.39 (-0.52, -0.28) | <.0001 | 0.85 (0.81, 0.89) | -1.97 (-2.68, -1.32) | <.0001 | 0.78 (0.73, 0.83) | | -0.32 (-0.42, -0.23) | <.0001 | 0.78 (0.72, 0.83) | | -0.99 (-1.31, -0.71) | <.0001 | 0.83 (0.78, 0.88) | | -0.25 (-0.34, -0.16) | <.0001 | 0.83 (0.77, 0.90) | | -2.00 (-2.98, -1.11) | <.0001 |
| Hainan | 0.61 (0.58, 0.65) | -0.07 (-0.08, -0.06) | <.0001 | 0.86 (0.84, 0.89) | -0.05 (-0.07, -0.04) | <.0001 | 0.91 (0.86, 0.95) | -0.19 (-0.30, -0.10) | <.0001 | 0.80 (0.75, 0.85) | | -0.05 (-0.06, -0.03) | <.0001 | 0.79 (0.74, 0.84) | | -0.14 (-0.18, -0.10) | <.0001 | 0.82 (0.76, 0.87) | | -0.04 (-0.06, -0.03) | <.0001 | 0.91 (0.85, 0.98) | | -0.16 (-0.30, -0.03) | 0.02 |
| Hebei | 0.58 (0.53, 0.63) | -0.73 (-0.89, -0.59) | <.0001 | 0.84 (0.77, 0.91) | -0.57 (-0.87, -0.28) | <.0001 | 0.81 (0.76, 0.88) | -3.41 (-4.72, -2.09) | <.0001 | 0.68 (0.62, 0.75) | | -0.66 (-0.86, -0.46) | <.0001 | 0.79 (0.72, 0.87) | | -1.22 (-1.78, -0.66) | <.0001 | 0.82 (0.75, 0.89) | | -0.37 (-0.55, -0.20) | <.0001 | 0.88 (0.79, 0.99) | | -1.88 (-3.72, -0.12) | 0.04 |
| Heilongjiang | 0.33 (0.32, 0.34) | -0.66 (-0.69, -0.63) | <.0001 | 0.44 (0.43, 0.45) | -1.08 (-1.12, -1.04) | <.0001 | 0.70 (0.68, 0.72) | -2.92 (-3.16, -2.67) | <.0001 | 0.60 (0.58, 0.61) | | -0.45 (-0.49, -0.41) | <.0001 | 0.76 (0.74, 0.79) | | -0.72 (-0.82, -0.61) | <.0001 | 0.82 (0.78, 0.86) | | -0.19 (-0.23, -0.14) | <.0001 | 0.92 (0.88, 0.96) | | -0.70 (-1.04, -0.36) | <.0001 |
| Henan | 0.56 (0.54, 0.58) | -1.23 (-1.34, -1.12) | <.0001 | 0.86 (0.84, 0.88) | -0.81 (-0.96, -0.67) | <.0001 | 0.83 (0.81, 0.86) | -5.02 (-6.01, -4.16) | <.0001 | 0.68 (0.65, 0.71) | | -1.10 (-1.27, -0.95) | <.0001 | 0.75 (0.72, 0.78) | | -2.43 (-2.81, -2.10) | <.0001 | 0.86 (0.82, 0.90) | | -0.49 (-0.65, -0.34) | <.0001 | 0.86 (0.81, 0.90) | | -3.95 (-5.40, -2.68) | <.0001 |
| Hubei | 0.32 (0.30, 0.35) | -1.08 (-1.19, -0.98) | <.0001 | 0.64 (0.61, 0.66) | -1.26 (-1.40, -1.13) | <.0001 | 0.84 (0.80, 0.88) | -2.72 (-3.53, -1.92) | <.0001 | 0.81 (0.76, 0.86) | | -0.37 (-0.50, -0.26) | <.0001 | 0.83 (0.79, 0.87) | | -0.98 (-1.29, -0.69) | <.0001 | 0.87 (0.81, 0.92) | | -0.24 (-0.36, -0.13) | <.0001 | 0.92 (0.86, 1.00) | | -1.18 (-2.34, -0.06) | 0.04 |
| Hunan | 0.60 (0.57, 0.62) | -0.77 (-0.85, -0.70) | <.0001 | 0.87 (0.85, 0.90) | -0.51 (-0.61, -0.40) | <.0001 | 0.86 (0.83, 0.89) | -2.78 (-3.47, -2.07) | <.0001 | 0.71 (0.68, 0.74) | | -0.66 (-0.75, -0.57) | <.0001 | 0.79 (0.76, 0.82) | | -1.40 (-1.67, -1.12) | <.0001 | 0.85 (0.79, 0.91) | | -0.33 (-0.48, -0.18) | <.0001 | 0.80 (0.76, 0.84) | | -3.58 (-4.44, -2.68) | <.0001 |
| Jiangsu | 0.44 (0.41, 0.47) | -0.36 (-0.41, -0.32) | <.0001 | 0.74 (0.68, 0.79) | -0.33 (-0.42, -0.24) | <.0001 | 0.71 (0.65, 0.77) | -1.90 (-2.51, -1.33) | <.0001 | 0.66 (0.59, 0.73) | | -0.26 (-0.34, -0.18) | <.0001 | 0.66 (0.59, 0.74) | | -0.69 (-0.94, -0.47) | <.0001 | 0.72 (0.61, 0.83) | | -0.20 (-0.32, -0.10) | <.0001 | 0.78 (0.68, 0.88) | | -1.36 (-2.15, -0.62) | <.0001 |
| Jiangxi | 0.62 (0.58, 0.68) | -0.72 (-0.88, -0.57) | <.0001 | 0.89 (0.86, 0.92) | -0.44 (-0.58, -0.30) | <.0001 | 0.93 (0.89, 0.97) | -1.49 (-2.44, -0.56) | <.0001 | 0.76 (0.72, 0.81) | | -0.54 (-0.67, -0.42) | <.0001 | 0.96 (0.91, 1.00) | | -0.29 (-0.59, 0.00) | 0.05 | 0.86 (0.81, 0.91) | | -0.32 (-0.45, -0.19) | <.0001 | 1.05 (0.98, 1.13) | | 0.85 (-0.40, 2.07) | 0.17 |
| Jilin | 0.61 (0.58, 0.64) | -0.43 (-0.48, -0.38) | <.0001 | 0.86 (0.84, 0.88) | -0.31 (-0.36, -0.27) | <.0001 | 0.90 (0.87, 0.92) | -1.23 (-1.62, -0.88) | <.0001 | 0.70 (0.68, 0.73) | | -0.40 (-0.45, -0.35) | <.0001 | 0.83 (0.80, 0.85) | | -0.67 (-0.80, -0.55) | <.0001 | 0.91 (0.86, 0.97) | | -0.11 (-0.19, -0.04) | <.0001 | 0.87 (0.83, 0.91) | | -1.40 (-1.91, -0.93) | <.0001 |
| Liaoning | 0.51 (0.49, 0.52) | -0.33 (-0.34, -0.31) | <.0001 | 0.66 (0.64, 0.67) | -0.46 (-0.48, -0.43) | <.0001 | 0.80 (0.79, 0.82) | -1.28 (-1.43, -1.13) | <.0001 | 0.42 (0.41, 0.44) | | -0.44 (-0.46, -0.41) | <.0001 | 0.78 (0.76, 0.80) | | -0.45 (-0.50, -0.39) | <.0001 | 0.85 (0.81, 0.89) | | -0.10 (-0.13, -0.07) | <.0001 | 0.92 (0.89, 0.95) | | -0.47 (-0.66, -0.27) | <.0001 |
| Inner Mongolia | 0.51 (0.49, 0.54) | -0.58 (-0.64, -0.51) | <.0001 | 0.76 (0.73, 0.80) | -0.56 (-0.66, -0.45) | <.0001 | 0.80 (0.76, 0.85) | -2.32 (-2.97, -1.65) | <.0001 | 0.61 (0.58, 0.66) | | -0.50 (-0.59, -0.41) | <.0001 | 0.86 (0.80, 0.92) | | -0.52 (-0.78, -0.27) | <.0001 | 0.84 (0.77, 0.93) | | -0.18 (-0.29, -0.08) | <.0001 | 0.97 (0.89, 1.05) | | -0.33 (-1.13, 0.46) | 0.44 |
| Ningxia | 0.50 (0.46, 0.55) | -0.10 (-0.12, -0.08) | <.0001 | 0.89 (0.87, 0.92) | -0.04 (-0.05, -0.03) | <.0001 | 0.84 (0.80, 0.88) | -0.32 (-0.41, -0.24) | <.0001 | 0.78 (0.75, 0.82) | | -0.05 (-0.06, -0.04) | <.0001 | 0.79 (0.75, 0.83) | | -0.13 (-0.16, -0.10) | <.0001 | 0.88 (0.84, 0.93) | | -0.03 (-0.04, -0.01) | <.0001 | 0.98 (0.91, 1.05) | | -0.04 (-0.16, 0.09) | 0.58 |
| Qinghai | 0.73 (0.66, 0.80) | -0.05 (-0.07, -0.03) | <.0001 | 0.93 (0.89, 0.98) | -0.02 (-0.04, -0.01) | 0.002 | 0.86 (0.81, 0.91) | -0.26 (-0.37, -0.17) | <.0001 | 0.64 (0.59, 0.69) | | -0.07 (-0.09, -0.06) | <.0001 | 0.63 (0.60, 0.66) | | -0.22 (-0.26, -0.19) | <.0001 | 0.70 (0.65, 0.75) | | -0.06 (-0.08, -0.05) | <.0001 | 0.82 (0.75, 0.89) | | -0.30 (-0.45, -0.18) | <.0001 |
| Shaanxi | 0.50 (0.47, 0.52) | -0.65 (-0.71, -0.59) | <.0001 | 0.79 (0.77, 0.82) | -0.53 (-0.62, -0.44) | <.0001 | 0.77 (0.74, 0.80) | -3.14 (-3.75, -2.60) | <.0001 | 0.77 (0.72, 0.81) | | -0.36 (-0.44, -0.28) | <.0001 | 0.78 (0.74, 0.82) | | -0.97 (-1.22, -0.76) | <.0001 | 0.75 (0.70, 0.81) | | -0.37 (-0.49, -0.27) | <.0001 | 0.87 (0.81, 0.92) | | -1.62 (-2.45, -0.94) | <.0001 |
| Shandong | 0.57 (0.55, 0.60) | -1.14 (-1.25, -1.03) | <.0001 | 0.84 (0.81, 0.88) | -0.85 (-1.07, -0.62) | <.0001 | 0.87 (0.83, 0.90) | -3.80 (-4.93, -2.62) | <.0001 | 0.73 (0.68, 0.78) | | -0.83 (-1.02, -0.63) | <.0001 | 0.86 (0.82, 0.91) | | -1.24 (-1.72, -0.75) | <.0001 | 0.83 (0.78, 0.88) | | -0.54 (-0.73, -0.34) | <.0001 | 0.99 (0.92, 1.05) | | -0.34 (-1.95, 1.21) | 0.68 |
| Shanghai | 0.40 (0.39, 0.42) | -0.46 (-0.49, -0.43) | <.0001 | 0.77 (0.75, 0.80) | -0.37 (-0.43, -0.32) | <.0001 | 0.81 (0.78, 0.83) | -1.70 (-2.02, -1.41) | <.0001 | 0.21 (0.20, 0.22) | | -0.77 (-0.82, -0.72) | <.0001 | 0.68 (0.66, 0.71) | | -0.94 (-1.06, -0.82) | <.0001 | 0.57 (0.53, 0.61) | | -0.39 (-0.46, -0.33) | <.0001 | 0.83 (0.79, 0.87) | | -1.36 (-1.81, -0.97) | <.0001 |
| Shanxi | 0.50 (0.46, 0.54) | -0.42 (-0.49, -0.36) | <.0001 | 0.81 (0.75, 0.86) | -0.32 (-0.44, -0.22) | <.0001 | 0.75 (0.69, 0.81) | -2.23 (-2.97, -1.53) | <.0001 | 0.62 (0.56, 0.69) | | -0.38 (-0.49, -0.28) | <.0001 | 0.70 (0.63, 0.79) | | -0.85 (-1.17, -0.54) | <.0001 | 0.65 (0.55, 0.76) | | -0.33 (-0.50, -0.20) | <.0001 | 0.74 (0.65, 0.85) | | -2.13 (-3.26, -1.11) | <.0001 |
| Sichuan | 0.62 (0.60, 0.63) | -0.94 (-0.99, -0.89) | <.0001 | 0.86 (0.84, 0.87) | -0.71 (-0.81, -0.62) | <.0001 | 0.85 (0.83, 0.87) | -3.89 (-4.53, -3.29) | <.0001 | 0.80 (0.77, 0.83) | | -0.57 (-0.66, -0.48) | <.0001 | 0.80 (0.78, 0.83) | | -1.67 (-1.93, -1.42) | <.0001 | 0.89 (0.86, 0.92) | | -0.32 (-0.43, -0.21) | <.0001 | 0.91 (0.88, 0.95) | | -2.03 (-2.92, -1.21) | <.0001 |
| Tianjin | 0.40 (0.39, 0.42) | -0.16 (-0.17, -0.15) | <.0001 | 0.75 (0.73, 0.77) | -0.14 (-0.16, -0.13) | <.0001 | 0.89 (0.86, 0.91) | -0.33 (-0.40, -0.26) | <.0001 | 0.85 (0.83, 0.88) | | -0.05 (-0.06, -0.04) | <.0001 | 0.97 (0.93, 1.00) | | -0.03 (-0.06, 0.00) | 0.04 | 0.88 (0.85, 0.92) | | -0.03 (-0.05, -0.02) | <.0001 | 1.17 (1.13, 1.22) | | 0.41 (0.32, 0.49) | <.0001 |
| Tibet | 0.72 (0.64, 0.80) | -0.01 (-0.02, -0.01) | <.0001 | 0.90 (0.85, 0.94) | -0.01 (-0.01, 0.00) | <.0001 | 0.93 (0.88, 0.99) | -0.03 (-0.06, 0.00) | 0.01 | 0.87 (0.80, 0.95) | | -0.01 (-0.01, 0.00) | <.0001 | 0.61 (0.57, 0.64) | | -0.06 (-0.06, -0.05) | <.0001 | 0.75 (0.69, 0.80) | | -0.01 (-0.02, -0.01) | <.0001 | 0.90 (0.82, 0.98) | | -0.04 (-0.08, -0.01) | 0.01 |
| Xinjiang | 0.60 (0.57, 0.64) | -0.29 (-0.34, -0.24) | <.0001 | 1.02 (0.97, 1.07) | 0.03 (-0.04, 0.10) | 0.42 | 0.89 (0.85, 0.94) | -0.76 (-1.12, -0.39) | <.0001 | 1.04 (0.97, 1.11) | | 0.03 (-0.03, 0.08) | 0.31 | 0.75 (0.71, 0.81) | | -0.51 (-0.65, -0.38) | <.0001 | 1.11 (1.04, 1.19) | | 0.09 (0.03, 0.14) | 0.002 | 1.41 (1.30, 1.53) | | 2.29 (1.84, 2.71) | <.0001 |
| Yunnan | 0.70 (0.67, 0.72) | -0.44 (-0.50, -0.39) | <.0001 | 0.87 (0.85, 0.91) | -0.37 (-0.46, -0.27) | <.0001 | 0.84 (0.81, 0.88) | -2.55 (-3.16, -1.91) | <.0001 | 0.73 (0.70, 0.76) | | -0.47 (-0.54, -0.40) | <.0001 | 0.77 (0.74, 0.81) | | -1.24 (-1.48, -0.96) | <.0001 | 0.74 (0.68, 0.79) | | -0.46 (-0.59, -0.33) | <.0001 | 0.80 (0.76, 0.85) | | -2.89 (-3.67, -2.07) | <.0001 |
| Zhejiang | 0.59 (0.57, 0.61) | -0.67 (-0.72, -0.62) | <.0001 | 0.84 (0.82, 0.87) | -0.55 (-0.65, -0.46) | <.0001 | 0.89 (0.86, 0.93) | -1.96 (-2.62, -1.28) | <.0001 | 0.83 (0.80, 0.86) | | -0.34 (-0.42, -0.26) | <.0001 | 0.92 (0.88, 0.96) | | -0.51 (-0.80, -0.23) | <.0001 | 0.78 (0.74, 0.83) | | -0.42 (-0.53, -0.30) | <.0001 | 0.97 (0.92, 1.02) | | -0.56 (-1.41, 0.28) | 0.17 |
| Total | 0.56 (0.54, 0.58) | -15.91 (-16.98, -14.89) | <.0001 | 0.85 (0.83, 0.87) | -11.28 (-12.79, -9.79) | <.0001 | 0.86 (0.84, 0.88) | -54.19 (-64.24, -44.62) | <.0001 | 0.74 (0.72, 0.76) | | -11.19 (-12.51, -9.91) | <.0001 | 0.83 (0.80, 0.86) | | -21.27 (-25.09, -17.39) | <.0001 | 0.85 (0.82, 0.89) | | -5.96 (-7.69, -4.22) | <.0001 | 0.94 (0.90, 0.97) | | -22.01 (-35.25, -9.91) | 0.00 |

*Measured in millions

Note: Blue cells indicate a statistically significant decrease, while light blue cells represent a decrease that is not statistically significant; Orange cells indicate a statistically significant increase, while light orange cells represent an increase that is not statistically significant.
