# Supplementary figures and images for: Policy stringency during the COVID-19 pandemic and healthcare services utilization in China: An interrupted time-series analysis
Source: PLoS Med. 2026 Mar 26;23(3):e1004672. doi: 10.1371/journal.pmed.1004672 (PMC13043060; doi:10.1371/journal.pmed.1004672)

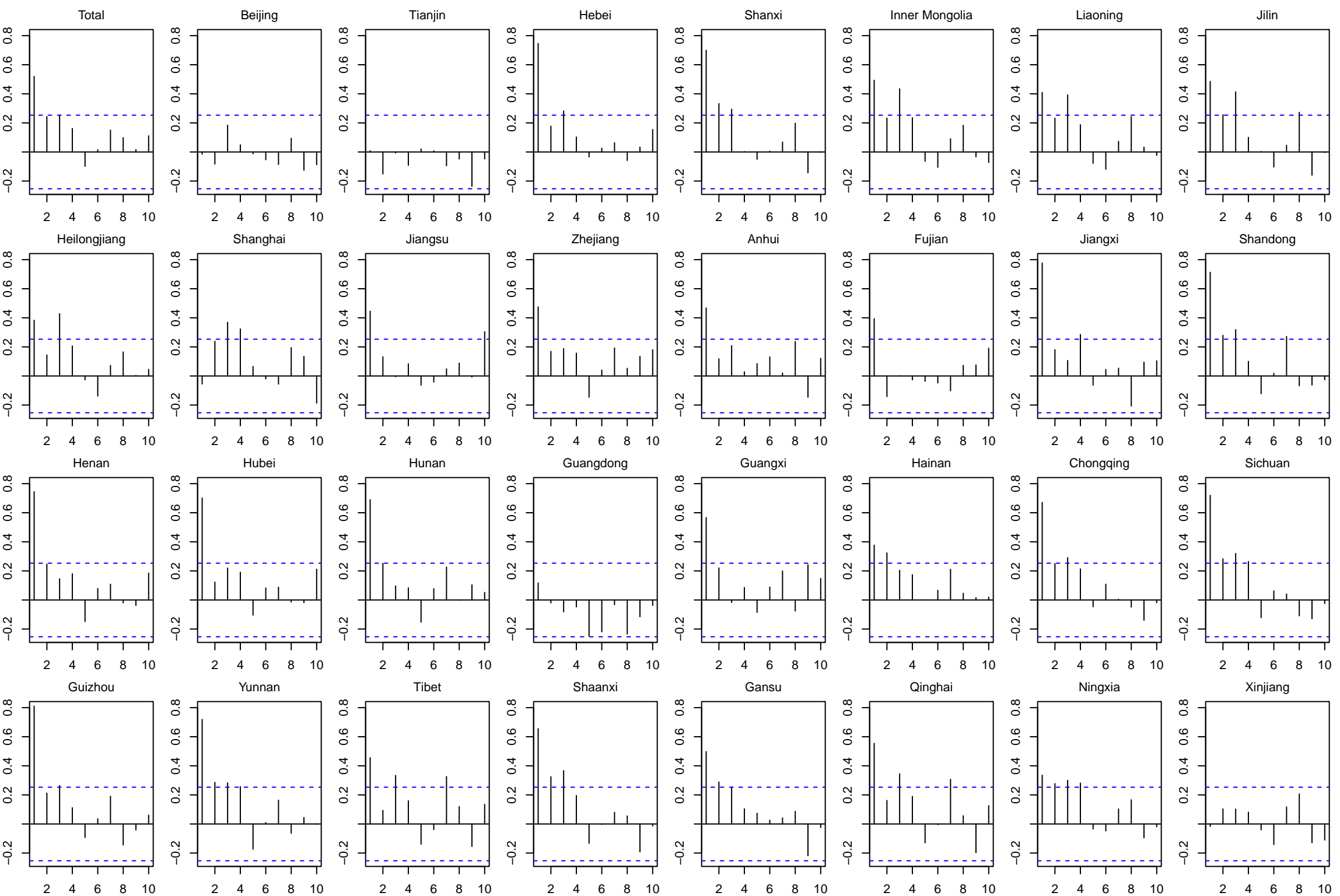

Supplement: S1 Fig — (PDF) [file pmed.1004672.s006.pdf]

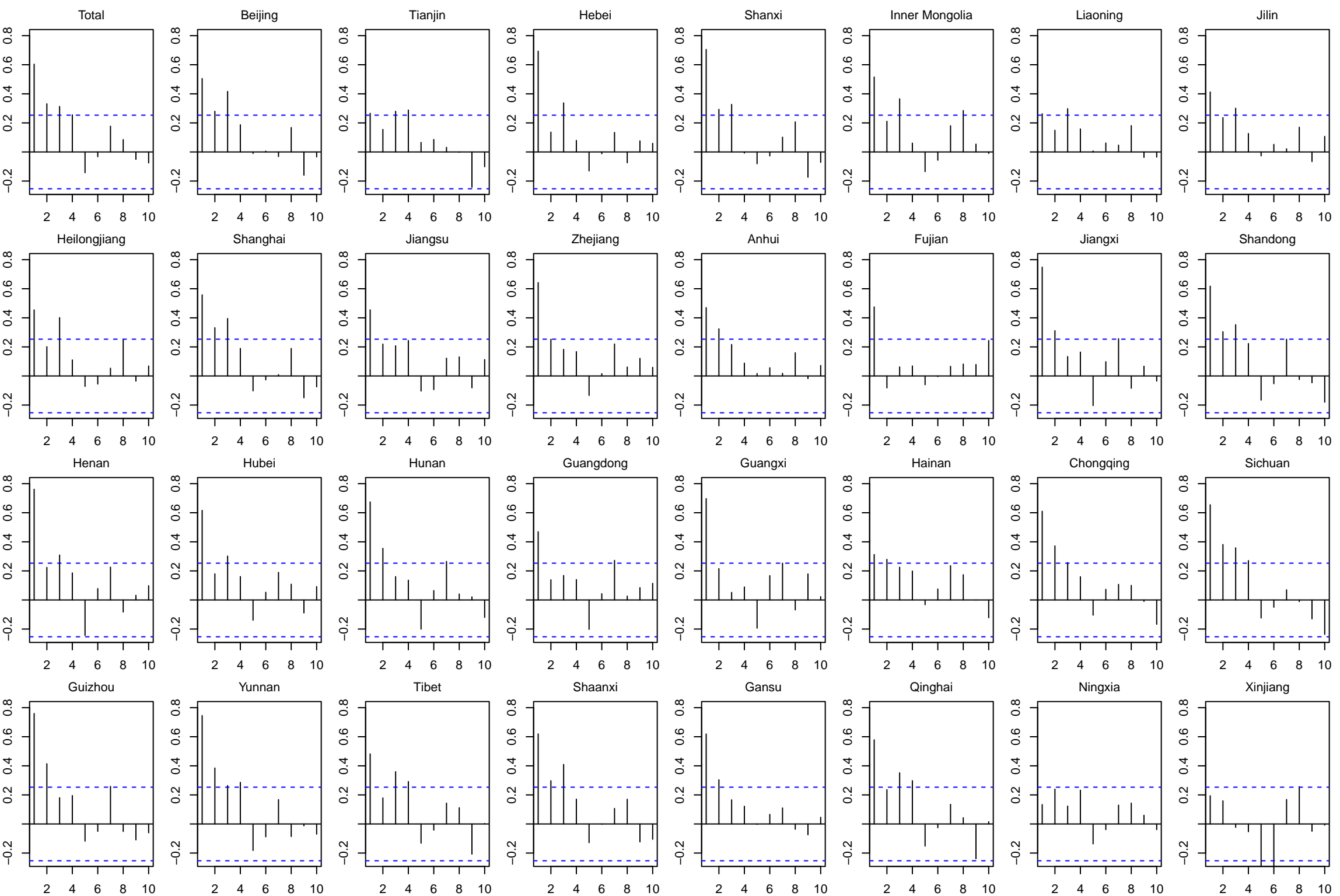

Supplement: S2 Fig — (PDF) [file pmed.1004672.s007.pdf]

Cumulative Loss in Hospital Visits per 1000-Person / 1000-Person-Month

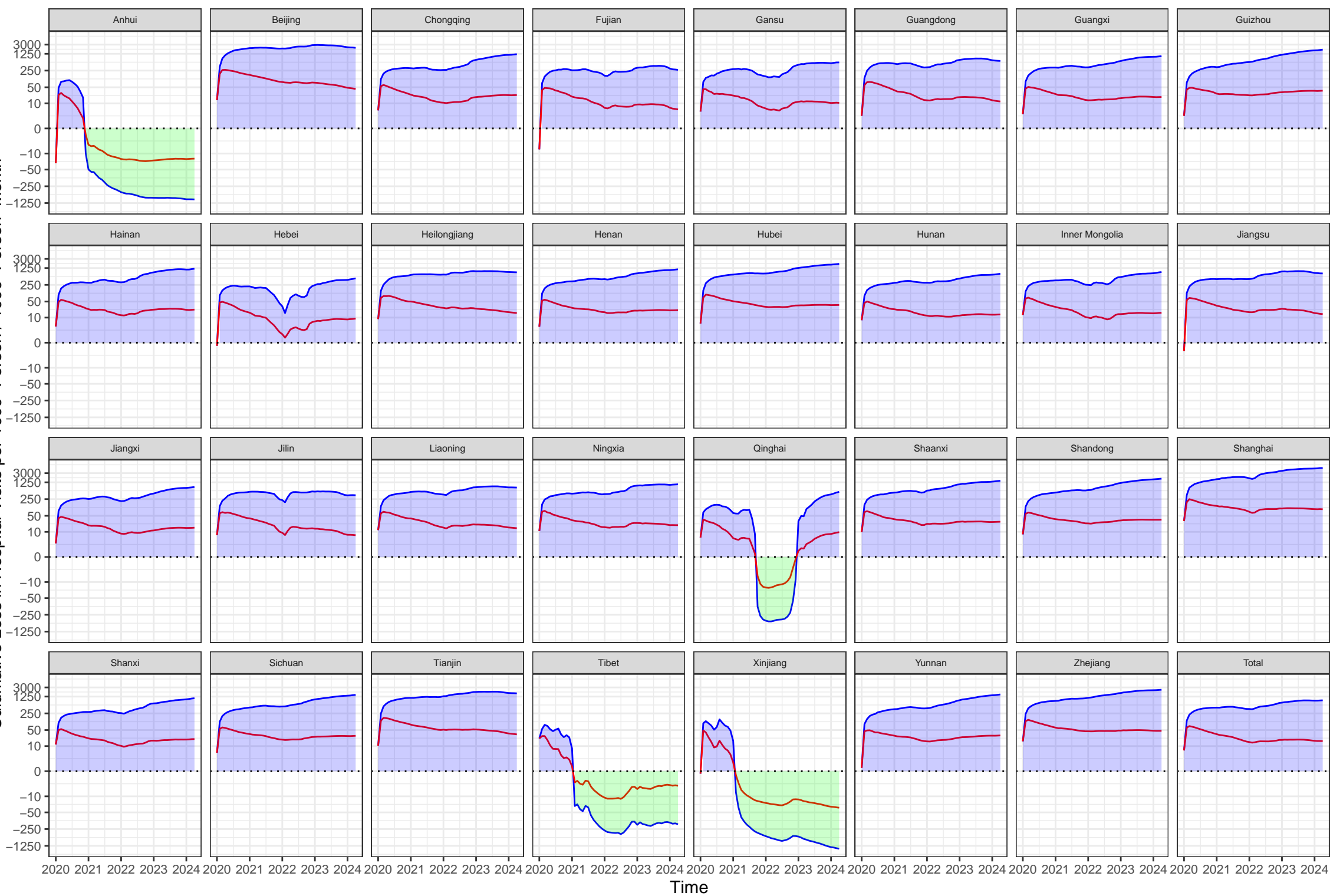

Supplement: S3 Fig — Cumulative loss is measured in number of visits per 1000-person (blue lines) and number of visits per 1000-person-month (red lines). (PDF) [file pmed.1004672.s008.pdf]

Cumulative Loss in Hospitalizations per 1000 Person / 1000-Person-Month

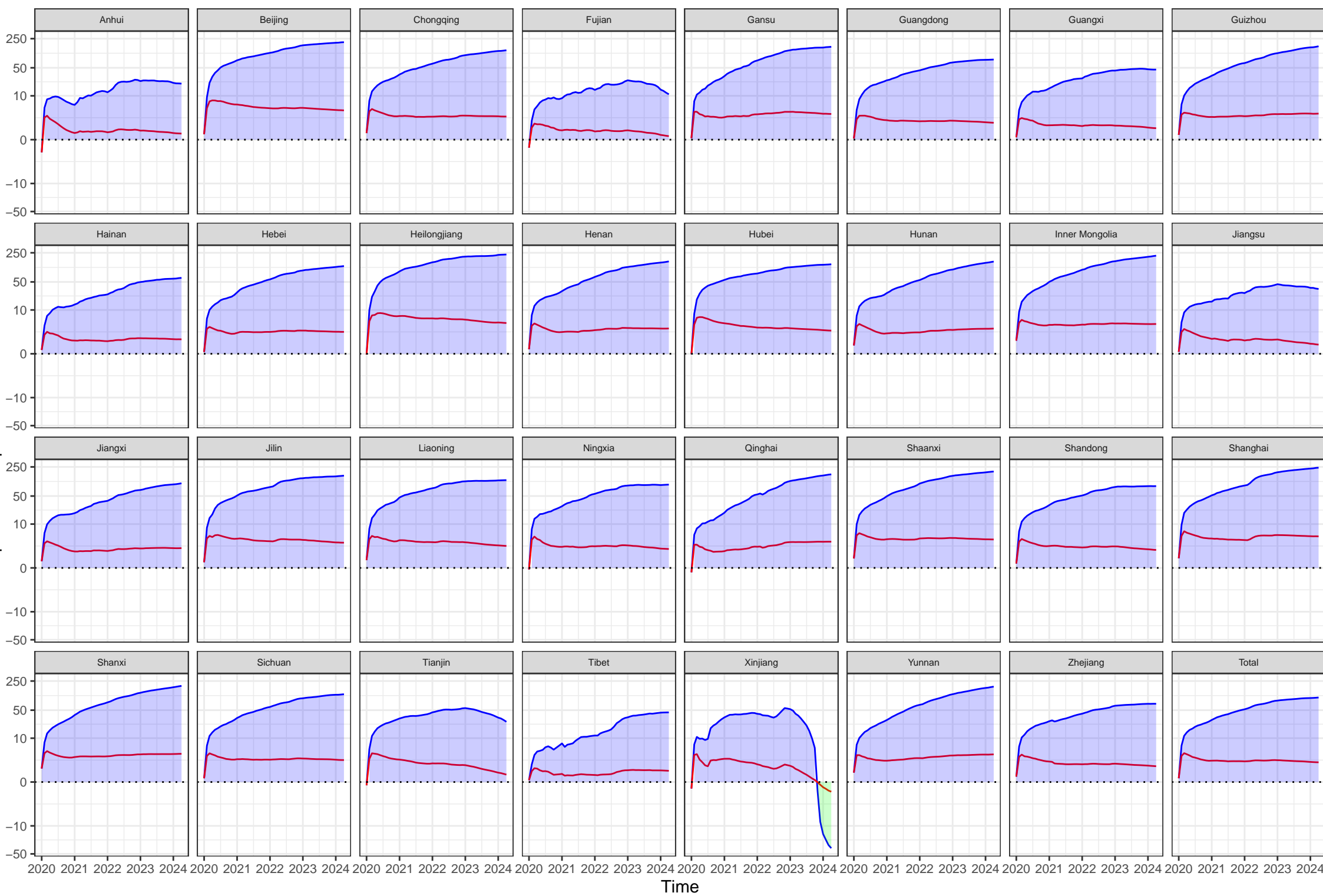

Supplement: S4 Fig — Cumulative loss is measured in number of visits per 1000-person (blue lines) and number of visits per 1000-person-month (red lines). (PDF) [file pmed.1004672.s009.pdf]

Ratio of Cumulative Observed to Cumulative Expected Utilization since January 2020

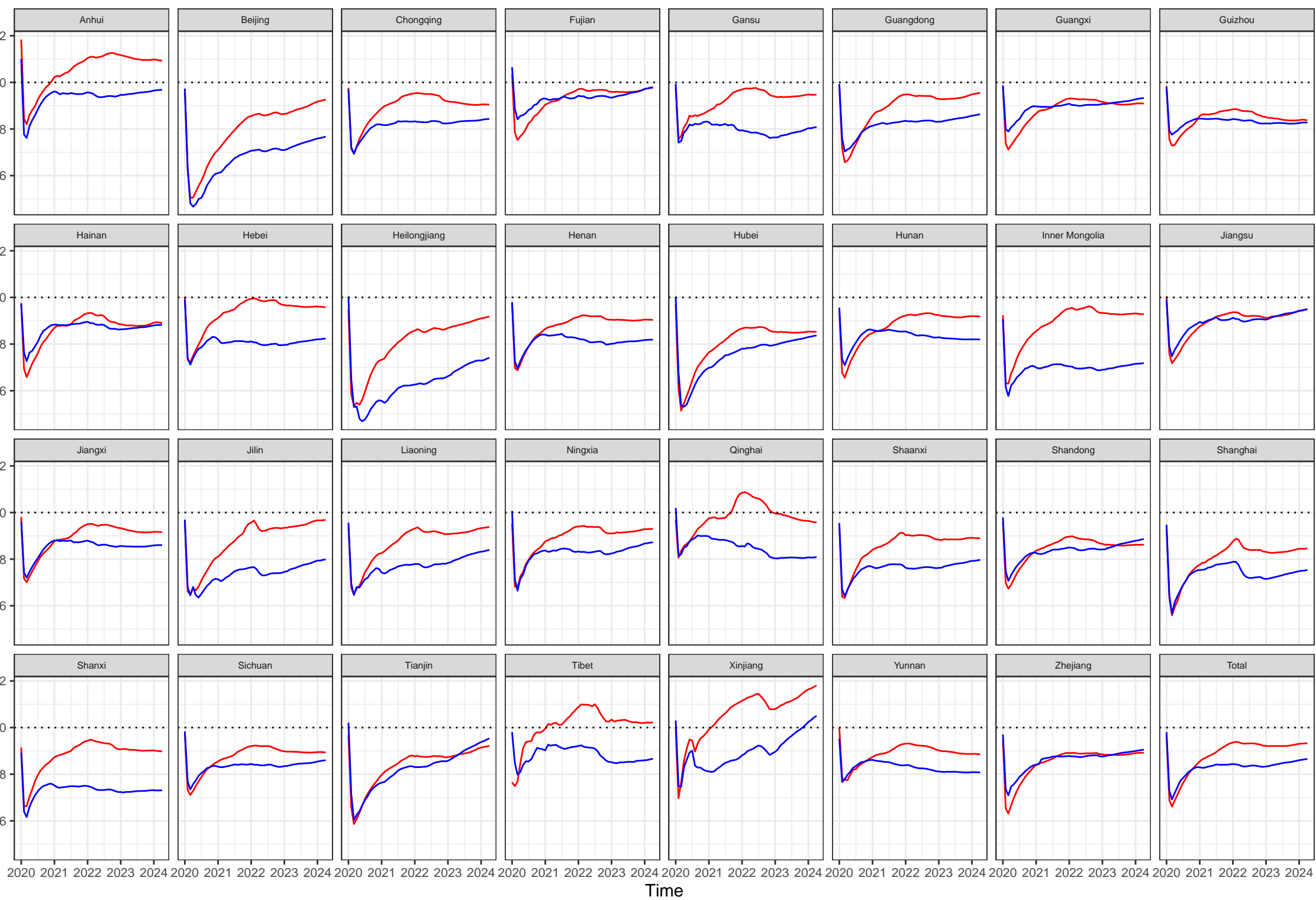

Supplement: S5 Fig — The red lines are for outpatient visits and the blue lines for inpatient discharges. (PDF) [file pmed.1004672.s010.pdf]
