# Supplementary material for: Policy stringency during the COVID-19 pandemic and healthcare services utilization in China: An interrupted time-series analysis
Source: PLoS Med. 2026 Mar 26;23(3):e1004672. doi: 10.1371/journal.pmed.1004672 (PMC13043060; doi:10.1371/journal.pmed.1004672)
Supplement: Attachment — Submitted filename: Response to Comments from the reviewers_final.docx [file pmed.1004672.s014.docx]

Till Winfried Bärnighausen, PhD

Editor-in-Chief

Andreia Cunha, PhD

Senior Editor

PLOS Medicine

Nov 2nd, 2025

# Dear Drs Bärnighausen and Cunha,

# We very much appreciate the insightful and constructive feedback from you and the reviewers regarding our manuscript, “The Incomplete Road to Recovery: Policy Stringency and Prolonged Impact of the COVID-19 Pandemic on Healthcare Services Utilization in China”. The comments have substantially improved the clarity and rigor of our work.

In response, we clarified our modeling strategy and provided additional justification for key analytic choices. Specifically, we explained the use of negative binomial regression to address over-dispersion in monthly counts and the application of Newey–West standard errors to account for residual autocorrelation. We also added visualizations of pre-pandemic monthly trends to support the stationarity assumption underlying our analyses.

We emphasized that changes in healthcare utilization during the pandemic were influenced by multiple factors beyond policy stringency, including healthcare supply, patient and provider behaviors, disease spectrum, and excess mortality. Reviewers noted that these time-varying factors could potentially act as confounders; in our analyses, we treated them primarily as intermediate pathways, reflecting their role in the causal sequence linking the pandemic to healthcare utilization. At the same time, our models included adjustments for long-term trends, seasonality, population changes, and key holidays to account for important time-varying factors not considered part of the causal pathway, thereby enhancing the robustness of our findings. Sensitivity analyses have confirmed the robustness of our findings, though we acknowledge limitations in disentangling pathway-specific effects.

To avoid causal overinterpretation, we revised the text throughout to ensure neutral, observational phrasing and to present results as descriptive trends and associations. The conclusions now highlight persistent changes in healthcare utilization during periods of high policy stringency while maintaining a neutral, observational tone.

Our specific changes are indicated below, with the reviewers’ comments in black, our responses in blue, and text from the manuscript that has been retained or revised, including new or modified text, underlined.

Sincerely,

Hong Xiao, PhD

Public Health Sciences Division

Fred Hutch Cancer Center

Email: xiaohongpku@gmail.com

**Comments from the reviewers:**

**Reviewer #1:** The paper presents an interrupted time series analysis of healthcare utilization in China after the onset of the COVID-19 pandemic. It is generally a well written and presented paper. I have the following comments regarding the statistical aspects of the work.

**1.1** Some further details on the justification for some modelling choices could be provided. The negative binomial regression model was chosen over a Poisson due to over-dispersion and likelihood ratio tests. Evidence of over-dispersion and the results of these likelihood ratio tests could be provided. Furthermore, the PACF could be shown to justify the use of Newey-West standard errors with three lags. After fitting the proposed model, was there any evidence of residual correlation?

**Response:** We thank the reviewer for this helpful statistical suggestion. Over-dispersion was evident as the variance of monthly counts exceeded the mean, and likelihood ratio tests strongly favored the negative binomial model over the Poisson (p < 0.001). The negative binomial model is more conservative in the presence of over-dispersion, yielding more robust inference than the Poisson model. The PACF of residuals indicated weak autocorrelation up to lag 3, motivating the use of Newey–West standard errors with three lags. Residual diagnostics showed no remaining serial correlation, confirming adequate model specification.

We have revised the method section accordingly:

“*Given a dispersed variation structure in monthly counts of utilizations and likelihood ratio tests favoring negative binomial regression over Poisson equivalents (p<0.001), we modeled the outcomes using a negative binomial regression*”

“*We applied Newey-West standard errors with up to three lags, per results of the PACF and Cumby-Huizinga general test, to address autocorrelation*.”

**1.2** Confidence intervals were produced using a parametric bootstrap approach. Why was this approach taken over using the asymptotic distribution of the model coefficients?

**Response:** We thank the reviewer for this question. To clarify, the confidence intervals were not generated using a parametric bootstrap as the reviewer suggested. Instead, we used the asymptotic distribution of the model coefficients as the reviewer suggested. Specifically, after fitting the model, we sampled 1,000 sets of coefficients from the asymptotic multivariate normal distribution defined by the estimated coefficients and their covariance matrix. These coefficient draws were then used to generate counterfactual monthly predictions, from which 95% confidence intervals were derived as the 2.5th and 97.5th percentiles. This approach provides a straightforward way to propagate coefficient uncertainty into predictions and their aggregated sums, while relying on the well-established asymptotic distribution of the estimated model parameters.

In response, we have revised the description of how absolute differences and confidence intervals were calculated to more clearly convey the approach. Specifically, the updated text now reads:

*“ Secondarily, we calculated absolute differences in outpatient visits and inpatient discharges. We generated 1,000 monthly counterfactual predictions by sampling from the multivariate normal distribution of the estimated model coefficients (i.e., based on their asymptotic distribution); the 2.5th and 97.5th percentiles defined 95% confidence intervals (CIs), and two‐sided P values were computed as twice the smaller proportion of simulations above or below zero.”*

We hope this revised wording more clearly conveys that the confidence intervals were derived from the asymptotic distribution of the model coefficients, addressing the reviewer’s insightful comment

**1.3**. The approach assumes a stationary distribution in the training period, that is the period considered before the COVID-19 pandemic. Visual inspection of the data, or of the residuals, could be presented to help to justify this assumption.

**Response:** We thank the reviewer for this helpful suggestion. Visualizations of the raw data are presented in Figures 1 and Figure 2 (Trends of observed and expected inpatient discharges per 1000 population per month), which support the assumption of a stationary distribution during the pre-pandemic period and justify the selection of linear regression for modeling.

**1.4**. The conclusion that the COVID-19 pandemic impacted healthcare utilisation assumes that there were no other factors that occurred during this time which could have also have affected healthcare utilisation. This could be considered in the discussion when interpreting the results. If there were other factors at play, could these have been included as covariates in the model to account for them?

**Response**: We agree with the reviewer that healthcare utilization during the COVID-19 pandemic may have been influenced by multiple concurrent factors. Our analysis did not assume that policy stringency was the sole determinant of healthcare utilization. Rather, the pandemic represents a broad exogenous shock that encompasses multiple mechanisms, including changes in healthcare supply, provider behavior, patient care-seeking patterns, disease spectrum, and even excess mortality among older adults—all of which could collectively contribute to the observed changes.

To account for time-varying factors independent of the pandemic, we included a linear term for long-term trends, fixed monthly effects to capture seasonality, and the number of Spring Festival days to account for its impact on hospital admissions (as described in the Methods section [Line 192-195]). Moreover, there were multiple indicators for COVID-19 landmarks, and the results of the association of these landmark exposures and outcomes were consistent with anticipated patterns and were highly interpretable, lending further validity to the results.

We discussed these potential mechanisms in the discussion section. However, incorporating them as separate covariates was not feasible because complete and reliable data were unavailable at the province–month level. Moreover, many of these factors are likely intermediate pathways through which the pandemic affected healthcare utilization, rather than independent confounders. Adjusting for them could therefore attenuate the overall estimated pandemic effect and obscure the total causal impact we aimed to quantify.

Based on the reviewer’s suggestion, we have added the following limitation in the discussion:

“*Fourth, our study could not fully disentangle the specific pathways through which the COVID-19 pandemic affected healthcare utilization, or to quantify the relative contribution of each factor. Multiple intermediary mechanisms, including changes in healthcare supply, provider and patient behaviors, disease spectrum, and mortality patterns may have been involved, but detailed data for pathway analyses were not available*.”

**Reviewer #2:**

This is a relevant and well-written article examining the impact of the COVID-19 pandemic on outpatient and inpatient care in China. While most studies focus on short-term effects, this analysis provides valuable insights by covering a longer period. It demonstrates that—assuming pre-pandemic secular trend in healthcare utilization—the effects of the pandemic persist at least through 2024, with significant regional variation. Furthermore, the study confirms a clear association between the stringency of public health measures adopted during the emergency and patterns of healthcare utilization.

Although the authors did not disaggregate the results by sex, age group, or health conditions, the article remains valuable for presenting aggregate data on healthcare activity during the pandemic, offering a clear summary of its overall impact on the health system. If these findings were discussed alongside data on healthcare spending, infrastructure, or human resources at the national level, they could yield valuable insights into healthcare productivity during this period—a highly relevant and often debated topic in recent years.

The results support the authors' claims, and the methods used align with those applied in similar studies, with only minor adaptations tailored to the specific context of this research. Particularly valuable are eFigure 1 and eFigure 2, which illustrate healthcare utilization trends throughout the study period, the model fitting for the pre-pandemic period, and counterfactual scenarios at both national and regional levels. With adequate access to the data, the results of the study could be easily reproduced.

I recommend accepting the manuscript with minor revisions.

**Response:** We thank the reviewer for the kind and encouraging feedback. We are delighted that our study and analyses are considered valuable, and we appreciate the suggestions provided.

**2.1** Line 459 - Consider expanding the color gradient to enhance visual differentiation of the pandemic's impact.
**Response:** We have expanded the color gradient in figures to improve visual differentiation of the pandemic’s impact across regions and periods. The revised figure now provides clearer contrast and enhances interpretability. Additionally, as suggested, we have moved eFigure 1 and eFigure 2 to the main manuscript because they provide informative insights.

**2.2** Line 463 - Please enlarge the graphic titles and legends. For graphics C and D, consider inverting the colors, using red to indicate decreases and blue for increases. Ensure this color scheme is clearly described in the figure legend.

Response: We have enlarged all graphic titles and legends as suggested. Regarding the color scheme for graphics C and D, we intentionally kept red (“hot”) to indicate increases and blue (“cold”) to indicate decreases to maintain consistency with the COVID-19 case color scheme. The figure legend has been updated to clearly describe this color convention.

**2.3** Line 473 - Please ensure that all figures are properly numbered (e.g., Figure A1, Figure A2). Also, consider inverting the colors—using red to indicate decreases and blue for increases—and ensure color usage is consistent throughout the article.

Response: We have reviewed and corrected all figure numbers and ensured that the color scheme is consistent throughout the article.

**2.4** Line 484 - Consider removing '02/2024-04/2024: Post Zero-COVID period.' Please enlarge the graphic titles and legends and reduce the size of the data points to better distinguish between color groups.

Response: We thank the reviewer for this suggestion. Although we considered removing the “02/2024–04/2024: Post Zero-COVID period”, we decided for now to retain it to remain consistent with the Methods and Results sections, as this period represents an important recovery phase. However, we defer to the consensus of the editor and reviewers regarding whether it is preferable to remove this.

We have also enhanced the color contrast for this period compared with other periods to make it more visually distinct. We have also enlarged the graphic titles and legends and reduced the size of the data points to better distinguish between color groups.

**Reviewer #3:**

Thanks for giving me the opportunity to review this interesting paper, but there are some major concerns for this manuscript.

Major concerns:
**3.1**. Causal claims exceed what the design supports. The manuscript repeatedly attributes declines and "incomplete recovery" in healthcare utilization to the Zero‑COVID policy (and to policy stringency). The analyses are ecological, aggregate interrupted time‑series with province-level stringency indices. This design cannot establish causality or apportion effects between policy, behavior change, healthcare supply constraints, mortality, demographic shifts, or other contemporaneous system changes. The language in the abstract, results, and discussion is causal/attributive and should be substantially toned down unless stronger causal identification is provided (e.g., credible quasi‑experimental contrasts, instrumental variables, or granular data linking policy actions to supply/demand changes).

The linear regression analysis associating the Policy Stringency Index and utilization is particularly weak as it does not adequately control for time‑varying confounders (e.g., case counts, health system capacity changes, economic shocks, mobility restrictions independent of formal policy index, telemedicine uptake). Without these, the negative association may simply reflect co-occurrence of outbreaks (high cases → high stringency → lower utilization) and not an independent effect of policy.

**Response:** We thank the reviewer for this important comment. We agree that, although interrupted time series (ITS) is a quasi-experimental design, our ecological, province-level analyses cannot definitively establish causality or disentangle the effects of policy, behavioral changes, healthcare supply constraints, or pandemic-related demographic shifts, all of which, in any event, are in the causal pathway of the occurrence of the pandemic itself.

Accordingly, we have revised the Abstract, Results, and Discussion to avoid causal or attributive language, replacing wording that could imply causation with neutral expressions emphasizing temporal or correlational relationships. For example, in the Introduction, we changed “influenced access to healthcare” to “*varied across different pandemic phases, including the Zero-COVID policy period and its cessation*.” Similarly, in the Results, we revised “were exacerbated by stringent Zero-COVID measures” to “*coincided with periods of stringent Zero-COVID measures*.” In the Conclusions, “The findings highlight the enduring consequences of stringent pandemic control measures on healthcare access” was changed to “*The findings highlight persistent changes in healthcare utilization observed during periods of stringent pandemic control measures*” to maintain a neutral, observational tone. Collectively, these revisions ensure that the manuscript accurately reflects an association-based interpretation of the observed trends rather than implying causation.

We further clarify that healthcare utilization during the COVID-19 pandemic was likely shaped by multiple concurrent factors. Our analysis does not assume that policy stringency was the sole determinant. Rather, the pandemic represents a broad exogenous shock affecting healthcare utilization through several mechanisms, including changes in healthcare supply, provider and patient behaviors, disease spectrum, and excess mortality; thus, all of these individual mechanisms are embedded within the causal pathway of the pandemic shock itself. The primary aim of our study was to describe overall temporal trends in healthcare utilization and their associations with key contextual indicators, rather than to quantify the contribution of specific pathways.

In addition, we note that in the Chinese context (illustrated in Figure 1), for a substantial portion of the study period, high policy stringency occurred in the absence of high case counts. Therefore, while co-occurrence is possible at other times, the observed temporal pattern provides some support for an independent association of policy stringency with healthcare utilization.

Based on the reviewer’s suggestion, we have added the following limitation in the discussion:

“*Fourth, our study could not fully disentangle the specific pathways through which the COVID-19 pandemic affected healthcare utilization, or to quantify the relative contribution of each factor. Multiple intermediary mechanisms, including changes in healthcare supply, provider and patient behaviors, disease spectrum, and mortality patterns may have been involved, but detailed data for pathway analyses were not available*.”

**3.2**. Incomplete control for time‑varying confounding and alternative explanations. The model controls for secular trend, month fixed effects, and Spring Festival days only. It does not sufficiently adjust for the most important time‑varying factors that plausibly affect utilization:

Local COVID incidence (using reliable case estimates) and healthcare burden (e.g., hospital occupancy, ICU strain). Mobility measures (internal travel data) and transport suspension events.
Supply‑side disruptions: temporary hospital/clinic closures, staff reallocation, suspension of elective services. Changes in population (excess mortality among older adults) and insurance coverage changes that the Discussion speculates about.

Authors used IHME estimates after official reporting stopped, but do not show sensitivity analyses to alternative case estimates or to omission of case counts. This omission weakens attribution to stringency.

Response: We thank the reviewer for these insightful observations.

As noted above, the primary aim of our study was to describe the overall impact of the COVID-19 pandemic on healthcare utilization, rather than to isolate or quantify the specific contribution of each pathway. Many of the time-varying factors that the reviewer suggested (e.g. COVID cases, mobility measures, supply disruptions) are best conceptualized as intermediate pathways through which the pandemic affected healthcare utilization, rather than independent confounders. Thus, since these factors are embedded within the causal pathway of the pandemic itself, adjusting for them could therefore attenuate the overall estimated pandemic effect and obscure the total impact we aimed to quantify.

Our models incorporated secular trends, month fixed effects, holidays, and population adjustments to account for demographic shifts (e.g., age structure and population growth). Consistent with prior studies, there was no substantial excess mortality in China before the lifting of the Zero-COVID policy, suggesting that major population losses were unlikely to confound our estimates.

COVID-19 case data were primarily used descriptively to illustrate the pandemic’s temporal trajectories. Importantly, as noted above, periods of high policy stringency often occurred independently of high case counts, indicating that stringency was not solely determined by local transmission intensity. For this reason, case counts, whether from official sources or IHME estimates, were not included in the regression models. Including them would have adjusted away part of the pandemic’s effect on healthcare utilization rather than clarifying it, as incident cases represent a component or consequence of the pandemic itself. Nonetheless, we verified that alternative case estimates from IHME follow broadly similar temporal patterns, and their omission is unlikely to materially alter the observed associations.

**3.3**. Problematic handling and imputation of stringency and missing data. The stringency index is imputed after Feb 28, 2023 by "decrementing 0.3

points per day until reaching zero." This is an arbitrary assumption with no justification and likely misrepresents policy heterogeneity in late 2022-2024. Such imputation could bias the stringency-utilization relationship and regional comparisons.

Missing province-level monthly utilization values were imputed as the mean of adjacent months; December 2022 was imputed from January 2023 due to "abrupt end of Zero‑COVID policy." These ad hoc imputations, especially around a key break point, are not acceptable without sensitivity analyses demonstrating robustness to alternative imputation methods (e.g., multiple imputation, interpolation with uncertainty, or excluding months with irregular reporting). The authors must provide diagnostics showing the extent and pattern of missingness and justify the imputation strategy statistically.

Response: We appreciate the reviewer’s thoughtful comments regarding the handling of missing stringency and utilization data. We emphasize that the stringency–utilization analyses did **not** include data from December 2022 onward, immediately after the abrupt lifting of the Zero-COVID policy, because policy volatility and regional heterogeneity during this transition period rendered direct temporal comparisons less meaningful. The analyses in Figure 3 therefore focus on the pre-lift period, where policy stringency and healthcare utilization were more stable and comparable.

The post-February 2023 decrement of 0.3 points per day was a pragmatic, descriptive approximation to reflect the observable, nationwide relaxation of restrictions, ensuring continuity in the visualization of long-term trajectories. This procedure was used solely for descriptive display and does not influence any inferential models or regional comparisons, all of which are restricted to empirically observed data prior to December 2022.

To clarify that the 0.3 decrement is not arbitrary, we have revised the Methods section to reflect:

*“Daily provincial stringency indices after February 28, 2023, were imputed by decrementing 0.3 points per day until reaching zero, based on the empirical distribution provincial index values and the subsequent timeline of key policy relaxation timeline.* [1-3]*”*

Regarding missing utilization values, we applied simple temporal averaging only to isolated months with incomplete reporting to maintain continuity in visualization. To ensure this approach did not influence our results, we conducted sensitivity checks by comparing trends and associations with and without these imputed points. These checks showed that excluding the imputed values had minimal impact on the overall patterns (Figure 1 and Figure 2). We acknowledge that alternative imputation approaches (e.g., multiple imputation, spline interpolation) could be explored in future work, but our analyses suggest that the main findings are robust to these limited imputations.

**3.4**. Interpretation of 'recovery' and 'unmet need' is unsupportable. The manuscript treats failure to return to pre‑pandemic utilization volumes as evidence of unmet need and healthcare inequity. This is not necessarily true: utilization may have become more appropriate (reduction of low‑value care), shifted to telemedicine, or been offset by changes in disease incidence (e.g., fewer injuries, less influenza) or demographic changes (excess mortality, declining births). The authors do not provide any outcome or quality data (mortality, emergency admissions for time‑sensitive conditions) to demonstrate harm from decreased utilization. Concluding that health needs are unmet is speculative.

To substantiate unmet need claims, authors would need linkage to health outcomes (excess mortality by cause, cancer stage at diagnosis, avoidable hospitalization rates) or at least present analysis by service type (elective vs. emergency; chronic disease follow‑up vs. preventive care).

Response: We thank the reviewer for this important comment. We agree that reduced healthcare utilization does not necessarily equate to unmet need, as declines may also reflect more appropriate care use, substitution by telemedicine, or changes in population health and demographics as the reviewer suggested. Our interpretation is constrained by the aggregate nature of available data, which lacks individual-level linkage to outcomes or quality indicators. Consequently, we could not assess changes in care quality or indirect health impacts, including quality of life, hospitalizations, or mortality. In addition, routine health system data did not capture telemedicine visits provided by third-party platforms. The examination of such aspects of this phenomenon, therefore, are beyond the scope of the available data, but are important to address in future analyses.

Our estimates of unmet need were based on counterfactual projections assuming no pandemic. However, actual healthcare needs may have changed due to shifts in environmental exposures, health behaviors, mental health, infectious disease prevalence, and demographic factors such as excess mortality among older adults and declining fertility. These factors could bias our estimates in either direction. Future research should integrate individual-level data to examine disparities in care use, evaluate quality and outcomes, assess the evolving role of telemedicine, and refine models of unmet need that incorporate demographic and epidemiologic change.

We have revised the Discussion to acknowledge these data limitations and to clarify that our findings should be interpreted within this context. The following text has been added:

“*Second, we could not assess changes in care quality or the broader indirect impact on health outcomes, including quality of life, hospitalization, and mortality. Third, routine health system data did not capture telemedicine visits provided by hospitals or third-party platforms*.”

“*Lastly, our estimates of unmet need were based on counterfactual projections assuming no pandemic. However, actual healthcare needs may have changed due to shifts in environmental exposures, health-related behaviors, mental health, infectious disease prevalence, and demographic factors such as excess mortality among older adults and declining fertility. These shifts may bias our estimates in either direction. Future research should integrate individual-level data to better examine disparities in healthcare use, assess care quality and outcomes, evaluate the evolving role of telemedicine, and improve models of unmet need that account for demographic and epidemiological changes*.”

Moreover, reductions in healthcare utilization may also reflect changes on the provider side rather than solely unmet patient need. These findings have implications for the long-term financial health of China’s healthcare system, including impacts on hospital revenue and provider behavior. To reflect this, we have added the following paragraph to the *Discussion* section:

*“These findings have potential implications for the long-term financial health of China’s healthcare system, including impacts on hospital revenue and health worker behavior. In China, most hospitals, including public ones, are largely self-financed through service charges and drug sales, with government subsidies accounting for only 9.7% to 11.7% of public hospital revenue in the Mideastern region, and slightly more in the northwest and north.[4], [5] During the early months of the pandemic, total healthcare expenditures dropped by 37.8% compared to the same period the previous year, based on 300 million bank card transactions.[6] Amid this revenue decline, reports emerged of salary reductions for health workers.[7, 8] Concerns have been raised that hospitals, facing financial strain, may have compensated by increasing the use of diagnostic tests and medical consumables. Despite an overall decrease in healthcare spending, per capita healthcare expenditures rose. [6] Shen et.al reported that in the first 25 weeks following the first wave in 2020, the average total expenses per patient rose by 8.7% to 16.7%, largely driven by higher laboratory test costs and medical consumables — most of which the additional expenses were reimbursed by health insurance. [9] The authors suggested these increases reflected widespread profit-compensation practices by hospitals in response to lost admissions during the initial COVID-19 wave.”*

**3.5**. Over-reliance on aggregate data without subgroup or service‑type analyses. Aggregate, hospital‑level monthly counts mask heterogeneity critical for interpretation. The inability to stratify by age, sex, admission type (elective vs emergency), specialty, or payer limits the value of conclusions about equity and policy implications.
The manuscript should either: (a) obtain and analyze more granular data (if available) to explore these distinctions, or (b) substantially hedge conclusions and present the work strictly as descriptive national/regional trends with clear limitations.

Response: We thank the reviewer for highlighting this important point. We note that these limitations have already been acknowledged in the manuscript. Specifically, we discuss that the use of aggregated, hospital-level monthly data precluded analyses stratified by individual characteristics such as sex, age, disease type, residency, insurance status, and socioeconomic factors. We also note that changes in care quality and broader health outcomes could not be assessed. We have carefully framed our conclusions to reflect these constraints, presenting the results primarily as descriptive national and regional trends.

“*This study has several limitations. First, the use of aggregated data precluded analysis by individual characteristics such as sex, age, disease type, residency, insurance status, and socioeconomic factors. Second, we could not assess changes in care quality or the broader indirect impact on health outcomes, including quality of life, hospitalization, and mortality*”

These revisions clarify that our findings should be interpreted within the context of available data and descriptive scope.

**3.6**. Use of case data and IHME estimates. The paper mixes official reported cases (until Nov 30, 2022) with IHME modelled estimates thereafter. The potential biases and uncertainty in IHME estimates—and their impact on analysis—are not discussed. Authors must perform sensitivity checks using alternative sources or explicitly quantify uncertainty introduced by using modeled case counts.

**Response:** COVID-19 case data were primarily used to illustrate the severity trends. As noted above, high policy stringency often occurred in the absence of high case counts, indicating that stringency is not entirely determined by case incidence.

Importantly, case counts were not included in the regression models, as incident cases are part of or a consequence of the pandemic. Therefore, while we included IHME modelled estimates to provide a more complete depiction of the pandemic trajectory in China after November 30, 2022, we do not expect their inclusion to materially affect our results or conclusions.

**3.7**. Substantially rewrite the claims to avoid causal language unless supported by stronger methods. Reframe the manuscript as descriptive/associational unless authors can provide stronger identification.

**Response:** We have carefully revised the manuscript to avoid causal language and have reframed it as descriptive and associational. All wording that could imply causation has been replaced with neutral expressions emphasizing temporal or correlational relationships. These revisions ensure that the manuscript accurately reflects an observational, association-based interpretation of the trends observed.

**Minior comments:**
**3.8.** Several figures are difficult to interpret (tiny thumbnails, unclear labels, confusing color schemes). The supplemental reviews suggested plotting case counts and utilization on a single graph for illustrative provinces—this would be useful. Figures that drive the main causal assertions (stringency vs utilization) must be clearer and transparent.

Regression outputs (coefficients, IRRs, dispersion) should be presented in main tables, not only narrative text.

**Response:** We thank the reviewer for the helpful suggestions. We have refined our figures to improve readability by enlarging them, clarifying labels, and making color schemes consistent. We also thank the reviewer for the suggestion of plotting case counts and utilization on a single graph. However, under China’s Zero-COVID policy, the association between case counts and healthcare utilization is relatively weak and therefore not the primary focus of our study. Instead, we visualized monthly case counts by province and month to illustrate the overall trends of case incidence and pandemic severity, while the main analyses focus on the association (rather than the causal relationship) between policy stringency and utilization. All figures supporting these analyses have been revised for improved clarity and transparency.

Regarding regression outputs, as suggested, we have presented IRRs, 95% CIs, and p-values in Table 2 for the entire study period by country, and in eTables 4 and 5 for pre-specified periods. These tables provide the primary regression results, while figures support data visualization and interpretation.

**3.9.** Numerous typographical and formatting errors throughout (e.g., "ublizabon" etc. in the submitted PDF); these suggest insufficient editorial polish.
Response: We have carefully reviewed the manuscript and corrected all identified errors to ensure proper formatting and editorial polish.

**3.10.** Some claims in the Discussion (e.g., large declines in insurance enrollment with specific numbers) are referenced to press articles or non‑peer reviewed sources. Prefer peer‑reviewed evidence or official statistics; otherwise clearly label as speculative or anecdotal.

Clarify definitions: what counts as outpatient visits (includes emergency?), inpatient discharges (does this include deaths during admission?), hospital levels included (all hospitals? private clinics?).
**Response:** We thank the reviewer for this helpful observation. In response, we have supplemented press and non–peer-reviewed sources with peer-reviewed evidence where available. Specifically, we now cite:

Gruber J, Lin M, Yang H, Yi J. China’s Social Health Insurance in the Era of Rapid Population Aging. JAMA Health Forum. 2025;6(4):e251105. doi:10.1001/jamahealthforum.2025.1105

Outpatient visits in our study include both routine outpatient and emergency visits, as noted in the Methods: “*For this study, we obtained data on monthly hospital visits (outpatient and emergency) and inpatient discharges from January 2015 to April 2024*.”

Inpatient discharges include all patients discharged from the hospital, regardless of whether they survived or died during admission. “*Hospitals at all levels, both public and private, report monthly healthcare utilization data to provincial platforms, where it is verified, aggregated, and submitted to the National Health Commission for quality control*.” This includes public and private hospitals that routinely report utilization data to the national system.

**3.11.** Explain whether inpatients who died shortly after admission were included; reviewer comments raised this point and it affects interpretation of hospitalization volume.
Clarify the time window used to declare a province "not recovered" (three consecutive months Feb-Apr 2024 below expected). Why three months? Is this robust to choosing two or four months?
**Response:** We thank the reviewer for raising these important points. All inpatients were included in our hospitalization counts, regardless of whether they died shortly after admission. This approach reflects the total inpatient utilization and avoids underestimating hospitalization volume.

Regarding the definition of a province as “not recovered,” we used three consecutive months below expected utilization (February–April 2024) to balance short-term fluctuations and sustained trends. While alternative windows (e.g., two or four months) could be considered, we selected three months as a pragmatic choice to identify meaningful deviations from expected utilization.

**Reference**

1.	The Lancet Regional Health-Western Pacific, *The end of zero-COVID-19 policy is not the end of COVID-19 for China.* Lancet Reg Health West Pac, 2023. **30**: p. 100702.

2.	Burki, T., *WHO ends the COVID-19 public health emergency.* Lancet Respir Med, 2023. **11**(7): p. 588.

3.	The State Council of the People’s Republic of China. *Notice on Adjusting COVID-19 Prevention and Control Measures*. 2023 [cited 2025 Oct 27 2025]; Available from: <https://www.gov.cn/zhengce/2023-04/12/content_5751088.htm>.

4.	Xu, J., et al., *Reforming public hospital financing in China: progress and challenges.* BMJ, 2019. **365**: p. l4015.

5.	Zhang, J., et al., *The heterogeneous impacts of government on health system efficiency in China's new health reform, 2010-2019.* Int Health, 2024. **16**(3): p. 357-367.

6.	Zhang, Y.N., et al., *Reduction in healthcare services during the COVID-19 pandemic in China.* BMJ Glob Health, 2020. **5**(11).

7.	*Covid-19: China’s ‘exhausted’ doctors and nurses work long hours for reduced pay*. 2023 [cited 2025 February 6]; Available from: <https://www.maravipost.com/covid-19-chinas-exhausted-doctors-and-nurses-work-long-hours-for-reduced-pay/>.

8.	Ye, Y. *China’s Medics Were Paid Less During the Pandemic, Survey Says*. 2020 [cited 2025 February 6]; Available from: <https://www.sixthtone.com/news/1005378>.

9.	Shen, C., et al., *Evaluating the Impact of COVID-19 on Hospital Profit Compensation Activities: A Difference-in-Differences Event Study Analysis in China.* Healthcare (Basel), 2023. **11**(9).
