# Supplementary material for: Policy stringency during the COVID-19 pandemic and healthcare services utilization in China: An interrupted time-series analysis
Source: PLoS Med. 2026 Mar 26;23(3):e1004672. doi: 10.1371/journal.pmed.1004672 (PMC13043060; doi:10.1371/journal.pmed.1004672)
Supplement: Attachment — Submitted filename: Response.docx [file pmed.1004672.s015.docx]

**03/02/2026**

* Please revise the last sentence of the funding statement to read: The funders had no other role in study design, data collection and analysis, decision to publish, or preparation of the manuscript.

Response: We have revised the funding statement as suggested.

“Research reported in this publication was supported by the Public Health Sciences Division of the Fred Hutch Cancer Center and “2022 Zhejiang Province High-Level Talent Training Program - Medical Rising Star Fund Support”. Hong Xiao and Joseph M Unger were researchers funded by Fred Hutch Cancer Center, Yuechong Cui was a researcher funded by “2022 Zhejiang Province High-Level Talent Training Program - Medical Rising Star Fund Support”. The funders had no other role in study design, data collection and analysis, decision to publish, or preparation of the manuscript.”

* PLOS Medicine requires that the de-identified data underlying the specific results in a published article be made available, without restrictions on access, in a public repository or as Supporting Information at the time of article publication, provided it is legal and ethical to do so. Please see the policy at <http://journals.plos.org/plosmedicine/s/data-availability>  and FAQs at <http://journals.plos.org/plosmedicine/s/data-availability#loc-faqs-for-data-policy>

Reponse: We have updated the Data sharing statement:

All data used in this study are publicly available (Health service data: <https://www.nhc.gov.cn/mohwsbwstjxxzx/new_index.shtml>; Policy Stringency Index: <https://www.bsg.ox.ac.uk/research/covid-19-government-response-tracker>; COVID cases: <https://www.healthdata.org/research-analysis/health-topics/covid> ). The minimal dataset underlying the findings reported in this article, together with relevant documentation, is available in the GitHub repository: <https://github.com/xiaohongpku/healthservice2015to2024> .

* PLOS defines the “minimal data set” to consist of the data set used to reach the conclusions drawn in the manuscript with related metadata and methods, and any additional data required to replicate the reported study findings in their entirety. Authors do not need to submit their entire data set, or the raw data collected during an investigation. Please submit the following data:

The values behind the means, standard deviations and other measures reported;

The values used to build graphs;

The points extracted from images for analysis."

Reponse: In accordance with PLOS Medicine’s data availability policy, we have provided the minimal dataset necessary to replicate the reported study findings in their entirety, including the values underlying the summary statistics and figures presented in the manuscript, along with relevant metadata and documentation. These materials have been deposited in a public repository: https://github.com/xiaohongpku/healthservice2015to2024. We have updated the Data Availability Statement accordingly.

* The Data Availability Statement (DAS) requires revision. For each data source used in your study:

a) If the data are freely or publicly available, note this and state the location of the data: within the paper, in Supporting Information files, or in a public repository (include the DOI or accession number).

Response: The data used in this study are publicly available. The locations of the original data sources are described in the manuscript (References 1, 18, and 20), and links to these sources have been added. The minimal dataset underlying the reported findings has been deposited in a public repository at: https://github.com/xiaohongpku/healthservice2015to2024.

b) If the data are owned by a third party but freely available upon request, please note this and state the owner of the data set and contact information for data requests (web or email address). Note that a study author cannot be the contact person for the data.

Response: Not applicable.

c) If the data are not freely available, please describe briefly the ethical, legal, or contractual restriction that prevents you from sharing it. Please also include an appropriate contact (web or email address) for inquiries (again, this cannot be a study author).

Response: Not applicable.

**02/25/2026**

* Please revise your title to comply with PLOS Medicine's style; we would suggest using the following: Policy stringency during the COVID-19 pandemic and healthcare services utilization in China: an interrupted time-series analysis

Response: We have revised the title as suggested to comply with PLOS Medicine’s style.

* Please revise the mention of 'first study' from the author summary and 'significantly' from the discussion when not applied to statistics.
Response: We have removed the term “first” from the Author Summary. We also replaced non-statistical uses of “significant(ly)” with more appropriate wording (e.g., “substantial(ly)” / “notable”) and reserved “statistically significant” only for results supported by statistical testing.

* Please replace the section heading Background for Introduction.

Response: We have replaced the section heading “Background” with “Introduction,” as suggested.

* Please revise the funding statement to say 'no other role' rather than 'no role.'

Response: We have revised the funding statement as suggested.

“Research reported in this publication was supported by the Public Health Sciences Division of the Fred Hutch Cancer Center and “2022 Zhejiang Province High-Level Talent Training Program - Medical Rising Star Fund Support”. Hong Xiao and Joseph M Unger were researchers funded by Fred Hutch Cancer Center, Yuechong Cui was a researcher funded by “2022 Zhejiang Province High-Level Talent Training Program - Medical Rising Star Fund Support”. The funders had no other role in study design, data collection and analysis, decision to publish, or preparation of the manuscript.”

* Please provide a legend for figure 5 in addition to a title.

Response: We have added a detailed legend for Figure 5 (including explanations of symbols/colors/abbreviations, as applicable) in addition to the figure title.

* Please check that the Capital of Statistics (COSX) community website maps' usage rights are compatible with CC BY licensing.

Response: Thank you for this important comment. We did not use any third-party map images; all figures were generated in R from administrative boundary shapefiles. To ensure compliance with PLOS’s CC BY requirements, we replaced the boundary dataset previously obtained via COSX with an alternative source that is distributed under the MIT License, which permits reuse and redistribution and is compatible with publication under CC BY with appropriate attribution. We regenerated the maps and documented the new data source (including the repository and access date) and its license in the figure captions.

“Maps were generated in R using provincial boundary shapefiles obtained from GitCode (accessed February 25, 2026; MIT License). Available from: <https://gitcode.com/open-source-toolkit/19fe0>”

* Your study is observational and therefore causality cannot be inferred. Please remove language that implies causality, i.e. terms such as impact or effect, and replate it by describing associations instead.

Response: Thank you for this important comment. We agree that causal interpretation should be made cautiously in an observational study. We used an interrupted time-series design as a quasi-experimental approach to estimate changes in healthcare utilization associated with policy phases/stringency, but we acknowledge that residual confounding and concurrent factors may affect causal interpretation. Accordingly, we have revised the manuscript to avoid overstating causality and to use more conservative language (e.g., “association,” “change,” or “was associated with”) where appropriate.

* For all observational studies, in the manuscript text, please indicate when reported analyses differ from those that were planned, transparent explanations for differences that affect the reliability of the study's results. If a reported analysis was performed based on an interesting but unanticipated pattern in the data, please be clear that the analysis was data-driven.

Response: Thank you for this guidance. We have revised the statement as follows “Although this observational study was not formally preregistered, all reported analyses were planned a priori based on the study objectives, available data, and the interrupted time-series design. No analyses were added in response to unanticipated patterns in the data (i.e., no data-driven/post hoc analyses were conducted), and no material deviations from the initial analysis plan occurred. Any minor technical refinements (e.g., model specification or sensitivity checks) are explicitly noted in the Methods section along with rationale”

**02/05/2026**

**Requests from Editors:**

GENERAL EDITORIAL REQUESTS
* Please revise your title to follow PLOS Medicine's style. Your title must be nondeclarative and not a question. It should begin with main concept if possible. "Effect of" should be used only if causality can be inferred, i.e., for an RCT. Please place the study design ("A randomized controlled trial," "A retrospective study," "A modelling study," etc.) in the subtitle (ie, after a colon).

Response: The title follow PLOS Medicine’s title.

* Please confirm that your abstract complies with our requirements, including format (three sections: Background, Methods and Findings, and Conclusions) and providing all the information relevant to this study type <https://journals.plos.org/plosmedicine/s/submission-guidelines#loc-abstract> 
Response: The abstract complies with the journal’s requirements

* Please ensure that the Introduction ends with a clear description of the study question or hypothesis.
Response: Introduction ends with a clear description of the study question.

* Please ensure that all abbreviations are defined at first use throughout the text.
Response: All abbreviations are defined at first use throughout the text.

* Please confirm that all numbers presented in the abstract are present and identical to numbers presented in the main manuscript text.

Response: Yes, all numbers presented in the abstract are present and identical to those in the main manuscript.

GENERAL
* Please review your text for claims of novelty or primacy (e.g. 'for the first time') and remove this language. In addition, please check that any use of statistical terms (such as trend or significant) are supported by the data, and if not please remove them.

Response: We have reviewed the manuscript accordingly and removed claims of novelty or primacy, as well as any unsupported statistical terminology.

* Please remove the 'conclusions' subheading from the discussion. Please also remove any other subheadings from the discussion.
Response: We have removed the “conclusions” or any other subheading from the discussion

* Statistical reporting: Please revise throughout the manuscript, including tables and figures.
- Please report statistical information as follows to improve clarity for the reader ""22% (95% CI [13,28]; p</=)"".
- Please separate upper and lower bounds with commas instead of hyphens as the latter can be confused with reporting of negative values.
- Please repeat statistical definitions (HR, CI etc.) for each set of parentheses."

Response: We have made the revision throughout the manuscript.

ABSTRACT
* In the last sentence of the Methods and Findings section, please describe the main limitation(s) of the study's methodology.

Response: We have added “Use of aggregated routine health system data limited individual-level analyses, assessment of care quality, and disentangling of causal pathways.”

* Please also include the important dependent variables that are adjusted for in the analyses.

Response: We have added “controlling for underlying secular trends and patterns”

* Please revise the last sentence of the Background to active voice (e.g. We comprehensively analyzed...).
Response: We have revised the last sentence of the Background to active voice as suggested:

“We comprehensively analyzed national trends in healthcare utilization over the first four years of the pandemic, examining how different phases and shifts in policy stringency were associated with access to and delivery of care across regions and service types, and exploring the relationship between policy stringency and healthcare utilization.”

* Please also define what the policy stringency index is in the Abstract for the general reader.

Response: We have added the brief definition.

* In the author summary, in the final bullet point of 'What Do These Findings Mean?', please include the main limitations of the study in non-technical language.
Response: We have added the main limitations as suggested

FUNDING STATEMENT
* The funding statement should include: specific grant numbers, initials of authors who received each award, URLs to sponsors’ websites. Also, please state whether any sponsors or funders (other than the named authors) played any role in study design, data collection and analysis, the decision to publish, or preparation of the manuscript. If they had no role in the research, include this sentence: “The funders had no role in study design, data collection and analysis, decision to publish, or preparation of the manuscript.”
Response: We have revise the statement as suggested.

“**Funding:** Research reported in this publication was supported by the Public Health Sciences Division of the Fred Hutch Cancer Center and “2022 Zhejiang Province High-Level Talent Training Program - Medical Rising Star Fund Support”. Hong Xiao and Joseph M Unger were researchers funded by Fred Hutch Cancer Center, Yuechong Cui was researcher funded by by “2022 Zhejiang Province High-Level Talent Training Program - Medical Rising Star Fund Support”. The funders had no role in study design, data collection and analysis, decision to publish, or preparation of the manuscript. **Grant number**- Not Applicable”

* It appears that one or more study authors is affiliated with one or more of the agencies that funded the study. Thus, the statement “The funders had no role in study design, data collection and analysis, decision to publish, or preparation of the manuscript” does not apply. Please revise the Financial Disclosure accordingly, as in "[Author name] is [author's role] at [funding agency]. The funders had no other role in study design…..”

Response: We have revise the statement as suggested.

“**Funding:** Research reported in this publication was supported by the Public Health Sciences Division of the Fred Hutch Cancer Center and “2022 Zhejiang Province High-Level Talent Training Program - Medical Rising Star Fund Support”. Hong Xiao and Joseph M Unger were researchers funded by Fred Hutch Cancer Center, Yuechong Cui was researcher funded by by “2022 Zhejiang Province High-Level Talent Training Program - Medical Rising Star Fund Support”. The funders had no role in study design, data collection and analysis, decision to publish, or preparation of the manuscript. **Grant number**- Not Applicable”

COMPETING INTERESTS STATEMENT
* All authors must declare their relevant competing interests per the PLOS policy, which can be seen here: <https://journals.plos.org/plosmedicine/s/competing-interests> For authors with ties to industry, please indicate whether any of the interests has a financial stake in the results of the current study.

DATA AVAILABILITY
* PLOS Medicine requires that the de-identified data underlying the specific results in a published article be made available, without restrictions on access, in a public repository or as Supporting Information at the time of article publication, provided it is legal and ethical to do so. Please see the policy at

<http://journals.plos.org/plosmedicine/s/data-availability> 

and FAQs at

<http://journals.plos.org/plosmedicine/s/data-availability#loc-faqs-for-data-policy>

* The Data Availability Statement (DAS) requires revision. For each data source used in your study:

a) If the data are freely or publicly available, note this and state the location of the data: within the paper, in Supporting Information files, or in a public repository (include the DOI or accession number).

Response: All data used are publicly available. The location of the data is stated within the paper.

b) If the data are owned by a third party but freely available upon request, please note this and state the owner of the data set and contact information for data requests (web or email address). Note that a study author cannot be the contact person for the data.

c) If the data are not freely available, please describe briefly the ethical, legal, or contractual restriction that prevents you from sharing it. Please also include an appropriate contact (web or email address) for inquiries (again, this cannot be a study author).

FIGURES

* Please provide titles and legends for all figures and tables (including those in Supporting Information files). Please define all acronyms used in each figure or table in its corresponding legend.

Response: All titles and legends for all figures and tables are provided

* Please ensure that where relevant figures include 95% CIs.

Response: N/A

* Please consider avoiding the use of red and green in order to make your figure more accessible

Response: Red and Green are not presented in the same figure

* Please confirm that the appropriate usage rights apply to the use of this map. Please see our guidelines for map images: <https://journals.plos.org/plosmedicine/s/figures#loc-maps>
Response: Maps were generated in R using the shapefile of provincial boundaries from the Capital of Statistics (COSX) community website (https://uploads.cosx.org/2009/07/chinaprovinceborderdata_tar_gz.zip).

OBSERVATIONAL, COHORT, CROSS-SECTIONAL, AND CASE CONTROL STUDIES

* Please ensure that the study is reported according to the STROBE guideline, and include the completed STROBE checklist as Supporting Information.  Please add the following statement, or similar, to the Methods: ""This study is reported as per the Strengthening the Reporting of Observational Studies in Epidemiology (STROBE) guideline (S1 Checklist).""

The STROBE guideline can be found here: <http://www.equator-network.org/reporting-guidelines/strobe/>
When completing the checklist, please use section and paragraph numbers, rather than page numbers."

Response: The statement has been added and the updated checklist were uploaded.

* Did your study have a prospective protocol or analysis plan? Please state this (either way) early in the Methods section.

a) If a prospective analysis plan (from your funding proposal, IRB or other ethics committee submission, study protocol, or other planning document written before analyzing the data) was used in designing the study, please include the relevant prospectively written document with your revised manuscript as a Supporting Information file to be published alongside your study, and cite it in the Methods section. A legend for this file should be included at the end of your manuscript.

b) If no such document exists, please make sure that the Methods section transparently describes when analyses were planned, and when/why any data-driven changes to analyses took place. 

c) In either case, changes in the analysis-- including those made in response to peer review comments-- should be identified as such in the Methods section of the paper, with rationale."

Response: we have added “No formal prospective protocol or analysis plan was prepared for this study. The primary analyses were pre-specified by the investigators before accessing the data. Any post-hoc or exploratory analyses conducted after examining the data are clearly indicated in the Methods and Results sections.”


* Your study is observational and therefore causality cannot be inferred. Please remove language that implies causality and refer to associations instead.

Response: We have revised accordingly.

* For all observational studies, in the manuscript text, please indicate: (1) the specific hypotheses you intended to test, (2) the analytical methods by which you planned to test them, (3) the analyses you actually performed, and (4) when reported analyses differ from those that were planned, transparent explanations for differences that affect the reliability of the study's results. If a reported analysis was performed based on an interesting but unanticipated pattern in the data, please be clear that the analysis was data-driven.

Response: This study tested the following pre-specified hypotheses: (1) was the COVID-19 pandemic associated with statistically significant changes in outpatient and inpatient healthcare utilization? (2) were stricter Zero-COVID policies, as measured by the Policy Stringency Index, associated with larger reductions in healthcare utilizations? (3) Has healthcare utilization recovered to pre-COVID levels as of April 2024? (4) Did the magnitude of changes in healthcare utilization vary across regions?

**Comments from Reviewers:**

**Reviewer #1:** I thank the authors for their response, which have generally covered my previous comments. It would be good to include, perhaps in the supplementary, the results which led to the discussed modelling choices. In line 192, a p-value is reported justifying the use of negative binomial regression, but it is not clear if this p-value is using the aggregated national data, or a selected province-level dataset. In addition, the PACFs and Cumby-Huizings results could be shown for each model fit (i.e., at the province-level) to support the chosen lags in the Newey-West standard errors.

Response: 1. For the values, we have changed “p<0.001” to “p<0.05 for both aggregated national data and provincial-level data”. 2. We thank the reviewer for the suggestion. We conducted PACF and Cumby-Huizinga diagnostics for representative provinces and found that the choice of lag length had minimal effect on model estimates. To maintain clarity, diagnostic plots by provinces are provided in Supplementary eFigure1 and eFigure. These results support the chosen lags. Sensitivity analyses using alternative lag lengths showed minimal impact on the estimates and conclusions, supporting the robustness of our findings.

**Reviewer #2:** This is a relevant and well-written article that summarizes the impact of the COVID-19 pandemic on outpatient and inpatient care. The period analyzed in this article is longer than in previous articles on this topic. It demonstrates that—assuming pre-pandemic secular trends in healthcare utilization—the effects of the pandemic persist at least through 2024, with regional variations. The manuscript has value for the global health literature because the case of China was highly particular, and the methods can be reproduced in other countries.

I recommend accepting the article without additional revisions.
Response: We thank the review for the comments.
